# Label2label: training a neural network to selectively restore cellular structures in fluorescence microscopy

**DOI:** 10.1242/jcs.258994

**Published:** 2022-02-10

**Authors:** Lisa Sophie Kölln, Omar Salem, Jessica Valli, Carsten Gram Hansen, Gail McConnell

**Affiliations:** 1 University of Strathclyde, Department of Physics, Glasgow G4 0NG, UK; 2 University of Edinburgh, Centre for Inflammation Research, Edinburgh EH16 4TJ, UK; 3 University of Edinburgh, Institute for Regeneration and Repair, Edinburgh EH16 4UU, UK; 4 Edinburgh Super Resolution Imaging Consortium, Institute of Biological Chemistry, Biophysics and Bioengineering, Heriot-Watt University, Edinburgh EH14 4AS, UK

**Keywords:** Convolutional neural networks, Content-aware image restoration, Antibody labelling, Noise2noise, Cellular structures, Fluorescence microscopy

## Abstract

Immunofluorescence microscopy is routinely used to visualise the spatial distribution of proteins that dictates their cellular function. However, unspecific antibody binding often results in high cytosolic background signals, decreasing the image contrast of a target structure. Recently, convolutional neural networks (CNNs) were successfully employed for image restoration in immunofluorescence microscopy, but current methods cannot correct for those background signals. We report a new method that trains a CNN to reduce unspecific signals in immunofluorescence images; we name this method label2label (L2L). In L2L, a CNN is trained with image pairs of two non-identical labels that target the same cellular structure. We show that after L2L training a network predicts images with significantly increased contrast of a target structure, which is further improved after implementing a multiscale structural similarity loss function. Here, our results suggest that sample differences in the training data decrease hallucination effects that are observed with other methods. We further assess the performance of a cycle generative adversarial network, and show that a CNN can be trained to separate structures in superposed immunofluorescence images of two targets.

## Abstract (precis)

**Summary:** Label2label is a new deep learning-based image restoration method that reduces cytosolic background signals in immunofluorescence images of cellular structures.

## INTRODUCTION

In recent years, deep learning has increasingly been used for image processing in cell biology (Belthangady and Royer, 2019). Specifically, convolutional neural networks (CNNs) with a U-Net architecture (Ronneberger et al., 2015) are employed for various tasks, from protein detection in transmission microscopy images (Christiansen et al., 2018; Ounkomol et al., 2018) to image segmentation of single cells (Falk et al., 2019) or specific cellular structures (Caicedo et al., 2019).

Fluorescence microscopy is a commonly used technique in cell biology for determining the spatial distribution and abundance of target proteins in cells. It relies on the use of highly specific labels to visualise the cellular components of interest. Fluorescent labels can be chemical stains, antibody labelling or molecular labelling, whereby cells are genetically altered to express fluorescent proteins (Suzuki et al., 2007). In this work, we mainly focus on immunofluorescence labelling in fixed cells.

Recently, CNNs were employed for content-aware image restoration (CARE) of corrupted fluorescence images. By training a U-Net with image pairs that were acquired with, for instance, different laser powers or exposure times, it was shown that a network was able to successfully restore denoised images of cell structures, such as the plasma membrane or the nucleus (Ronneberger et al., 2015; Weigert et al., 2018). CNNs were further used for the upsampling of images that were detected below the Nyquist sampling frequency (Weigert et al., 2018). Moreover, generative adversarial networks (GANs) were trained to enhance image resolution in immunofluorescence images, using, for example, stimulated emission depletion (STED) microscopy to acquire the training benchmarks (Goodfellow et al., 2014; Wang et al., 2019).

Both mentioned methods rely on clean benchmark images for the network training, which can be challenging to acquire in practice. Hence, semi-supervised/unsupervised deep learning-based restoration methods have emerged. For instance, networks based on the architecture of a cycle generative adversarial network (CycleGAN) (Zhu et al., 2017) were employed to deconvolve fluorescence images of microtubules, using unpaired high-resolution images as reference (von Chamier et al., 2021) or simulated low- and high-resolution images (Lim et al., 2020) for the training. The CycleGAN architecture addresses the ‘hallucination problem’, which is the introduction of artificial features in generated images that is often observed when training a classical GAN (Zhu et al., 2017). Here, a GAN is first trained to generate a higher quality image based on a corrupted input. Then, the generated image is fed into a second GAN that translates it back into the original image (back translation), giving the network less freedom to make changes to an input (Zhu et al., 2017; Lim et al., 2020). Other examples include noise2void, an unsupervised method that removes camera shot noise (Krull et al., 2019), and noise2noise (N2N) (Lehtinen et al., 2018). For N2N, a CNN is trained with corrupted image pairs of the same sample. Owing to the statistical nature of how loss is minimised by the network during training with a deviation-minimising loss function, it was shown that uncorrelated image signals that follow, for example, Gaussian, Poisson or Bernoulli noise are rejected, whereas correlating fluorescence signals in the training data are recovered by this method (Lehtinen et al., 2018).

Although these restoration methods enhance the contrast in immunofluorescence images by upsampling or denoising, they do not correct for inherent cytosolic background signals in the specimen itself, which, in immunofluorescence microscopy, originate from the cell label or labelling protocol. Unspecific labelling by a stain or antibody binding, as well as internalisation or residue dyes after the specimen preparation, can significantly limit the image contrast of a target (Saper, 2009). The performance of antibodies or stains vary for a number of reasons. Epitopes can be altered in the target protein by the fixation step, effectively changing the location or accessibility to the antibody (Miller and Shakes, 1995). Also, unspecific antibody binding can be caused by attractive intermolecular interaction, such as van der Waals or hydrogen bonding interactions, or by binding to proteins with similar epitopes, which overall results in an underlying cytosolic background signal in images of cells (Wagner et al., 2011).

### Label2label

We propose a new application of deep learning in fluorescence microscopy in which a neural network is trained to significantly reduce label-induced unspecific cytosolic signals in fluorescence images of cellular structures. We call this method label2label (L2L). For L2L, a CNN is trained with image pairs of cells that are dual-labelled for the same distinct cellular structure of interest. L2L utilises the varying performance of antibodies and stains in immunofluorescence microscopy. We hypothesized that a CNN trained with two images of a cellular structure that originate from two non-identical labels would act content filter-like, whereby fluorescence signals that systematically vary in the images are rejected, whereas correlating structural signal is restored. Here, the underlying principle of L2L is a so-called style transfer in which a neural network is trained to merge the content in input images with the style of reference images (Jing et al., 2020). As input and benchmark images highly correlate, L2L is also comparable to N2N. In both methods, a network is trained without clean benchmark images; however, in L2L, differences between the training images are not only originating from dynamic image corruptions like noise, but also inherent sample (=label) differences. Consequently, fluorescence signals from cytosolic protein and unspecific binding that, in practice, lower structural contrast in immunofluorescence images are retrieved in restorations after N2N training, whereas a network reduces such signals after L2L training when selecting appropriate training data.

L2L is also different to restoration methods with a noise2clean approach in which a clean benchmark is required to train a network (Goodfellow et al., 2014; Weigert et al., 2018; Wang et al., 2019). For noise2clean, the training data are generated by acquiring two images of the same label with, for example, different exposure time, sampling density or frame averaging. Notably, training a network to restore immunofluorescence images that are acquired with low exposure time or sampling density is rarely feasible, as cell specimens are fixed, and commercial antibodies are comparably efficient and photostable, allowing the image acquisition with both parameters optimised right away. Generating image pairs to train a network to restore images acquired with low frame averaging, however, is time-consuming, and further complicated by stage drift and photobleaching, overall resulting in a low benefit-cost ratio. More importantly, as in N2N, background signals originating from the label are still present in the benchmark, and as such they also cannot be corrected by this method.

For L2L, the image pairs for the network training can be acquired simultaneously, under near-identical imaging conditions (see the imaging sections in Materials and Methods). The images of one fluorescent cell label are selected as training input, and the images of the label that yields a higher contrast of the cellular structure are used as a training benchmark. We selected the CSBDeep framework for the training that is also known as CARE network (Weigert et al., 2018). Moreover, we trained a CycleGAN with unpaired images to assess whether, in principle, a network can also be trained with immunofluorescence images that stem from two different datasets (Zhu et al., 2017).

To establish and evaluate our method, we generated image data across four different distinct cellular structures: the actin cytoskeleton (Dugina et al., 2009; Suarez and Kovar, 2016); the microtubule network (Nogales, 2000; Pellegrini and Budman, 2005); and discrete plasma membrane structures, namely caveolae (Rausch and Hansen, 2020) and focal adhesions (Martino et al., 2018). The ability of a CNN to selectively restore distinct cellular structures in immunofluorescence images after training with carefully selected data was further assessed by training a CNN as separator of two markers in superposed immunofluorescence images. Here, images were acquired of fixed cells that were dual-labelled with a nuclear marker and an antibody against the plasma membrane protein CD44 (Ilangumaran et al., 2010).

### Loss functions for training a convolutional neural network

We trained a CNN with different loss functions with the aim of restoring cell images with enhanced structural contrast. A CNN, which can be described as a function *g_θ_* with its model parameters *θ*, is trained to minimise the error between two images based on a loss function *L*:
(1)

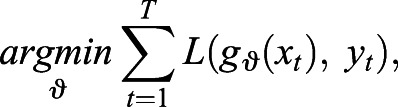
with *g_θ_*(*x_t_*) as the predicted image for the input *x_t_*, *y_t_* as the benchmark and *T* as the number of input-benchmark image pairs that are used for the training (Lehtinen et al., 2018).

Common loss functions are the least absolute deviation loss function *L_1_* and the least square deviation loss function *L_2_* (Zhao et al., 2016; Lehtinen et al., 2018):
(2)

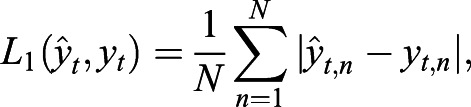

(3)

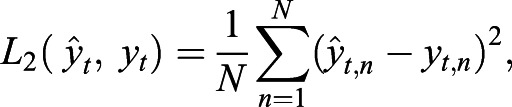
where 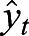 (=*g_θ_*(*x_t_*)) is the predicted image and *N* the total pixel number.

Because *L_2_* is minimal if it equals the mean value of the observations, it was used for N2N in cases in which the image corruption resembles, for example, Gaussian noise whose mean is zero (Lehtinen et al., 2018). *L_1_* is the loss function of the CSBDeep framework in default configuration (for non-probabilistic training). As for both, *L_1_* and *L_2_*, loss is calculated on a pixel-to-pixel basis during the network training, predicted images are often of low quality for a human observer (Larsen et al., 2016; Zhao et al., 2016).

To better take into account the properties of the human visual system, a multiscale structural similarity (MS-SSIM) index was proposed as an alternative to compare the similarity between two images (Wang et al., 2003, 2004). It follows:
(4)

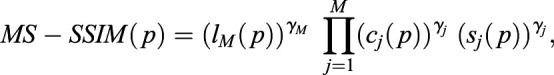
with the exponent *γ_j_* as the weight for the individual scale *j* 
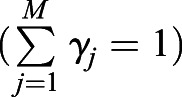
, and measures that compare the luminance (*l_M_*), contrast (*c_j_*) and structure (*s_j_*) between two images that are functions of the local statistics of a pixel *p* (for a detailed explanation see Wang et al., 2003, 2004). For the calculation, a low-pass filter is applied to the image patches after each iteration (if *M*>1), followed by downsampling by a factor of 2, making the MS-SSIM index sensitive to differing viewing conditions.

The MS-SSIM index exhibits values between (−1, 1) where −1/0/1 imply a negative/no/positive correlation between the images. To satisfy Eqn 1, for an MS-SSIM loss function (*L_MS-SSIM_*) follows (Zhao et al., 2016):
(5)

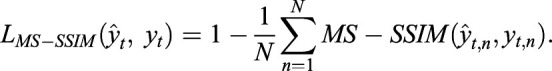
We hypothesized that a CNN would restore images with higher structural contrast after L2L training with a *L_MS-SSIM_* instead of a ‘classical’ *L_1_*. To test this hypothesis, we trained a CNN with different loss functions [*L_1_*, *L_SSIM_* (*M*=1), *L_3S-SSIM_* (*M*=3), *L_5S-SSIM_* (*M*=5)]. Weights of the MS-SSIM loss function were selected such that they follow the contrast sensitivity function of the human visual system (Wang et al., 2003). The results were compared to the denoising method N2N. For N2N training, the same settings were applied, but the network was trained with two noise realisations of the same label instead. The aim of this work is to compare two deep learning-based restoration methods that improve the contrast of cellular structures in fluorescence images, do not require clean benchmark data and have requirements for the training data generation that are feasible in standard immunofluorescence microscopy. We show proof-of-principle that by introducing systematic sample differences in the training data a CNN can be successfully trained to reject not only image noise but also diffuse label-dependent cytosolic signals in immunofluorescence images. Both can decrease the contrast of a target structure significantly in practice.

## RESULTS

### Label2label for reducing depolymerised β-actin in images of HeLa cells

**Fig. 1.**
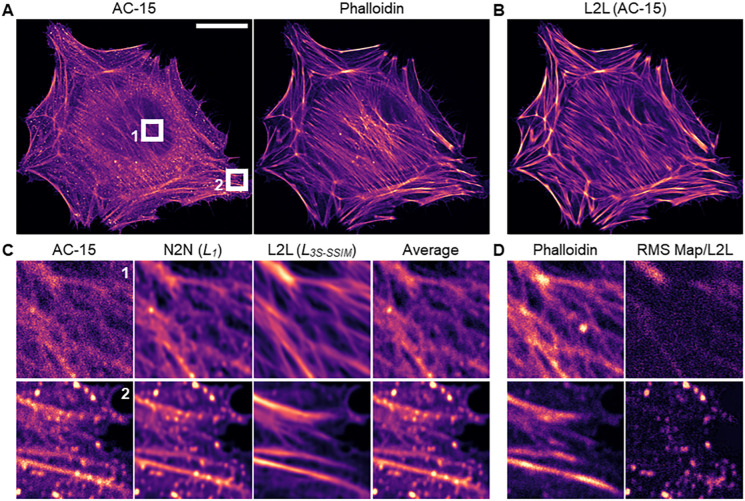
**Qualitative L2L and N2N results for images of actin.** (A) Confocal image pair of a fixed HeLa cell that was dual labelled with the anti-β-actin antibody AC-15 and a phalloidin stain, which was excluded from the CNN training. Scale bar: 20 µm. (B) Reconstructed image of AC-15 by a CNN after L2L training with images of AC-15/phalloidin as training input/benchmark, using an *L_3S-SSIM_* loss function. (C) Original and processed images of AC-15 for two ROIs (6 µm×6 µm). From left to right: raw image, restored images after N2N and L2L training with an *L_1_* and *L_3S-SSIM_* loss function, respectively, and a 20-frame average. (D) The corresponding image of phalloidin and the RMS map between the raw and the predicted image of AC-15 by the network after L2L training.

Image pairs were acquired of fixed HeLa cells (*N*=68) that were dual labelled with the monoclonal anti-β-actin antibody AC-15 and a phalloidin stain (see Materials and Methods). Fig. 1A shows an example confocal image pair of a fixed cell. Phalloidin almost exclusively labelled the actin filaments, but images of the antibody (AC-15) showed an additional high background signal in the cell body. This background signal likely originates from unspecific binding and/or binding to cytosolic protein by the antibodies, resulting in high intensity punctate regions, as observed in the cell cytoplasm. Notably, a 20-frame average image of AC-15 exhibited less image noise, but cytosolic background signal was still present, significantly lowering the contrast of the actin filaments (Fig. 1C). The difference in image contrast between both labels is quantifiable by calculating the mean Michelson and root mean square (RMS) contrast for each label (Peli, 1990). For that, we applied a Gaussian filter (sigma=2) to the images, normalised them to their 1st/99th percentile and derived the sample intensities assuming that the 10% brightest image pixels represented the sample. For the cell images of AC-15/phalloidin, mean Michelson contrast values of 0.44±0.08/0.96±0.04 and mean±s.d. RMS contrast values of 0.15±0.03/0.19±0.01 were calculated. Consequently, images of AC-15 were used as input and images of the phalloidin stain were used as a benchmark for L2L training. For N2N, two noise realisations of AC-15, acquired through sequential imaging, were used as training data.

Fig. 1B shows the restoration of an image of AC-15 in a HeLa cell (Fig. 1A, left) by a CNN after L2L training with a *L_3S-SSIM_* (see ‘Training the CNN’ section in Materials and Methods). A trained CNN reduced cytoplasmic signal throughout the cell body in the restoration, and the relative signal of filamentous actin labelled with AC-15 increased. In Fig. 1C, for two regions of interest (ROIs), the original cell image of AC-15 and the prediction by a CNN after N2N and L2L training with a *L_1_* and *L_3S-SSIM_*, respectively, are shown. Although noise was reduced in the N2N result, making it optically similar to a 20-frame average, the contrast of the actin filaments was still low due to the high background signal. In the L2L results, however, not only image noise was removed, but also the contrast of filamentous signal was clearly enhanced, even compared to the training benchmark (phalloidin) (Fig. 1D, left). The RMS maps between the raw images of AC-15 and the L2L results revealed a selective removal of high intensity punctate regions [Fig. 1D (right); for further maps see Fig. S1].

In Fig. S2A-C, the qualitative N2N and L2L results are shown for images of AC-15 dependent on the training loss function (*L_1_*, *L_5S-SSIM_*, *L_3S-SSIM_* or *L_SSIM_*), including the corresponding benchmarks (phalloidin) for L2L and 20-frame average images of both labels to better assess the network performance. For both methods, using an *L_1_* for the training leads in comparison to more conservative predictions, in which, with L2L, non-filamentous signal was reduced by the network but actin filaments appeared relatively blurry. On the other hand, predictions by a CNN after training with a *L_MS-SSIM_* showed cell structures with increased sharpness, and erroneous predictions by the network occurred (with lower *M*) (see annotated ROIs in Fig. S2A-C). Hallucination effects were substantially more pronounced in N2N results. Here, punctuate regions in the cell cytoplasm appeared with artificial contrast in restored images of AC-15, and actin filaments with low contrast in the input image were restored with intensity fluctuations along the structure. For L2L, these artefacts were barely observed when using an *L_MS-SSIM_* for the training.

To further evaluate the network performance after L2L training, the average peak signal-to-noise ratio (PSNR), normalised root-mean square error (NRMSE) and MS-SSIM indices (M=1, 3 and 5) were calculated for the raw or predicted images of AC-15, and the corresponding images of phalloidin, dependent on the training loss function. For that, validation image patches that were excluded from the training data were used (see the ‘Training the CNN’ section in Materials and Methods). All calculated metrics indicated an increased correlation between the restorations and the benchmark (phalloidin) compared to the original image (see Table 1). Notably, using an *L_5S-SSIM_* for the training narrowly yielded the best PSNR and NRMSE.

**Table 1.**
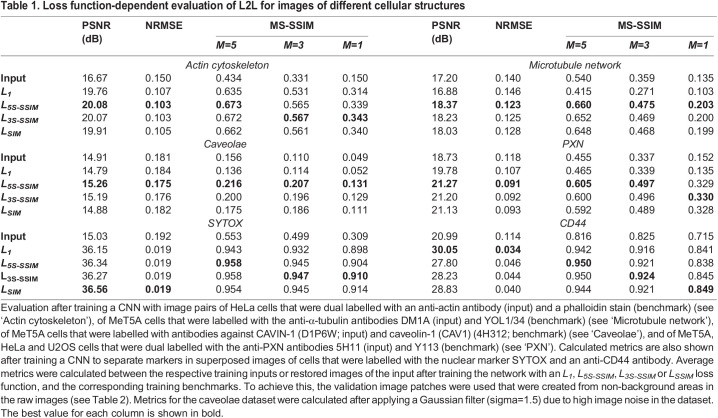
Loss function-dependent evaluation of L2L for images of different cellular structures

### L2L to enhance the structural contrast in images of the microtubule network and caveolae

To further study L2L as a method to increase image contrast of distinct cellular structures, fluorescence image pairs were acquired of the microtubule network that has a distinct branched spatial distribution in cells, and caveolae that are 60-100 nm large invaginations in the plasma membrane (Bates et al., 2007; Khater et al., 2018). For the former, fixed MeT5A cells were dual labelled with two monoclonal antibodies against α-tubulin (DM1A raised in mouse and YOL1/34 raised in rat), and confocal image pairs (*N*=51) were generated. To train a CNN as a content filter for caveolae, STED image pairs (*N*=60) were acquired of fixed MeT5A cells that were dual labelled with antibodies against the two essential caveolae components CAVIN-1 (D1P6W) and caveolin-1 (CAV1; 4H312) (Hansen and Nichols, 2010). For the tubulin dataset, we calculated mean±s.d. Michelson contrast values of 0.45±0.14/0.9±0.05 and mean±s.d. RMS contrast values of 0.13±0.03/0.17±0.02 for DM1A/YOL1/34; for the caveolae dataset, these values were 0.27±0.05/0.98±0.02 and 0.13±0.02/0.22±0.02 for D1P6W/4H312 (see previous section; for the caveolae dataset, the 1% brightest pixels in the image were regarded as a sample). Hence, for L2L training, images of DM1A and D1P6W were used as input, and images of YOL1/34 and 4H312 were used as a benchmark to train the CNN for the respective target structure.

In images of cells labelled with YOL1/34, intensity fluctuations along the microtubules were observed that likely originate from inhomogeneous binding or selective binding to specific epitopes of polymerised tubulin; this was not observable for the clone DM1A (compare 20-frame average images in Fig. S2D,F). Microtubules appear overall sharper in images of YOL1/34 compared to DM1A, with a lower ‘haze’ in the cytoplasm. This haze might be caused by unspecific binding, binding to cytosolic tubulin and/or out-of-focus signal. Notably, the optical resolution in images of DM1A labelled with the secondary antibody Alexa Fluor 633 was lower than in images of YOL1/34, which was conjugated to Alexa Fluor 488.

**Fig. 2.**
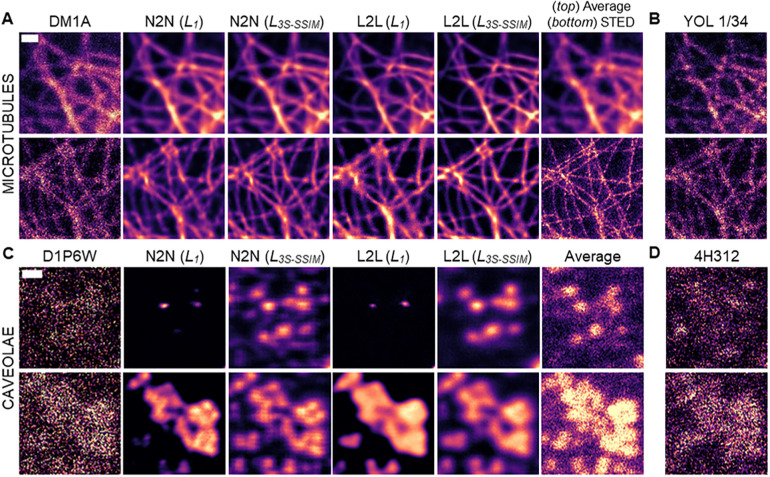
**Loss function-dependent L2L and N2N results for images of the microtubule network and caveolae.** (A-D) Confocal images of MeT5A cells that were dual labelled with the anti-tubulin antibodies DM1A (A) and YOL1/34 (B), and STED images of MeT5A cells that were dual labelled with the anti-CAVIN-1 antibody D1P6W (C) and the anti-CAV1 antibody 4H312 (D). (A,C) From left to right: raw image of a representative training input, reconstructed images after N2N and L2L training with an *L_1_* or *L_3S-SSIM_* loss function, and a corresponding 20-frame average or high resolution STED image. (B,D) Representative training benchmarks for L2L training are displayed. Images shown were excluded from the network training. Scale bars: 1 µm (A,B); 200 nm (C,D).

In Fig. 2A, the results after N2N and L2L training with the tubulin dataset are displayed for two ROIs and loss functions (*L_1_* and *L_3S-SSIM_*). Restorations are shown of a representative training input (top) and an image of the same cell specimen, which was acquired with a different microscope that allowed additional STED imaging (bottom) (see the imaging section in Materials and Methods). STED images exhibit a circa 3× enhanced resolution compared to the standard confocal image and are therefore a better reference for assessing the restoration performance of a CNN for this sample in ROIs in which microtubules are densely packed (Bates et al., 2007). Comparing the restorations by a CNN after ‘classical’ N2N training with an *L_1_* with the L2L result after employing an *L_3S−SSIM_* for the training, a clear improvement of the contrast of the microtubule network with L2L was observed (Fig. 2A). Here, both methods outperformed the corresponding 20-frame average image (Fig. 2A, top; Fig. S2D,F) and classical image processing methods, such as a Gaussian or top-hat filter (Fig. S3, Table S1). The restoration of the cell image of DM1A that was acquired with a different imaging setup than the training input exhibited a slightly less homogeneous intensity distribution along the microtubules, likely due to the changed image noise (compare Fig. 2A, left). For N2N and L2L, the restoration success is dependent on the tubule density in the image. The closer microtubules are packed in the cell, the less likely the successful recovery of separate structure by the CNN, as evident by comparing the results of both methods with the corresponding STED image (see also Fig. S4A,B). Notably, the intensity fluctuations along the cellular structure in the benchmark (YOL1/34) for L2L do not result in artefacts in restorations of the input (DM1A). Further N2N and L2L results for representative training inputs are shown in Fig. S2E, after using an *L_1_*, *L_5S-SSIM_*, *L_3S-SSIM_* or *L_SSIM_* for the training. A loss function-dependent trend was observable for both methods: using an *L_MS-SSIM_* instead of an *L_1_*, the CNN learned (with decreasing *M*) to restore microtubules with increased sharpness, especially when trained with images of two non-identical labels (Fig. S2E). This effect was quantifiable; extracted full width at half maxima (FWHMs) of line profiles across single microtubules in the images showed that results obtained with L2L were closest to the microtubule diameters detected with STED microscopy (Fig. S4C).

The STED image pairs that we acquired of cells that were dual-labelled for CAVIN-1 (D1P6W) and CAV1 (4H312) showed a low signal-to-noise ratio, further amplified by the pre-processing step that was undertaken to prevent artefacts that we observed when training with the nearly binary raw data (see ‘Data augmentation and pre-processing’ in Materials and Methods; Fig. 2C,D). Moreover, the correlation of the fluorescence signal originating from caveolae structure was relatively low between the training input (D1P6W) and benchmark (4H312) for some image pairs (Fig. S5A,C). This was also indicated by the calculated mean PSNR, NRMSE and MS-SSIM indices for both images, which suggested the lowest correlation among the L2L datasets that were generated in this work (compare Table 1). It posed the question as to whether a CNN would be prone to introduce artificial structure after L2L training with this dataset.

We found that the CNN performance after N2N and L2L training was highly dependent on the training loss function (Fig. 2C). Using an *L_1_*, in both methods, a trained CNN only learned to restore structures in high signal-to-noise regions in the input image (compare Fig. 2C, top and bottom). However, when the training loss function was replaced with an *L_3S-SSIM_*, the recovery of structural signal by the CNN from the noisy input (DM1A) was much enhanced for N2N and L2L. Here, cytosolic background signal in the image was clearly reduced by the network after L2L training, resulting in restorations with higher sample-to-background ratios than in corresponding 20-frame average STED images of both labels (Fig. 2; Fig. S5A-C). For N2N, on the other hand, significant hallucination effects were observed after using an *L_3S-SSIM_* for the training, with unspecific cytosolic signals often recovered as structure-like content (Fig. 2C, top). These artificial features got more pronounced when using the loss function with a small number of iterations (*M*) (compare N2N results for *L_SSIM_* and *L_5S-SSIM_* in Fig. S5B). Moreover, weak stripe-like artefacts were introduced by the network after N2N training (Fig. 2C). Although caveolae structures appeared slightly sharper than in the L2L results, the level of sharpness that was observed in the N2N result was not verifiable in the 20-frame average image (Fig. 2C, bottom).

Again, the network performance was evaluated after L2L training with different loss functions by calculating the average PSNR, NRMSE and MS-SSIM indices (Table 1). For both datasets, the calculated metrics indicated that the correlation between reconstructed and benchmark image was highest after training with an *L_5S-SSIM_*. For the tubulin dataset, a decrease in the correlation between the restored image of DM1A and the benchmark (YOL1/34) after training with a *L_1_* was observed.

### Training networks to reduce cytosolic content in images of PXN with paired and unpaired images

**Fig. 3.**
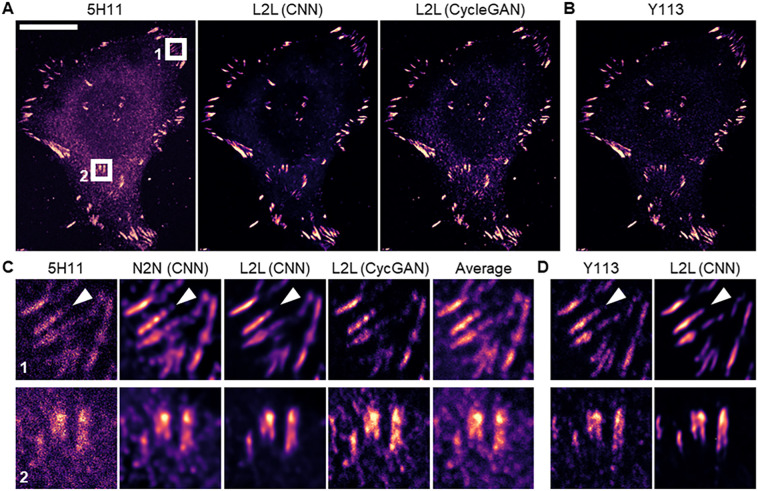
**Network architecture-dependent L2L results for images of PXN.** Confocal images of a HeLa cell dual labelled with the two anti-PXN antibodies 5H11 and Y113, which were used as training input and benchmark for L2L training. (A) From left to right: raw image of 5H11, the restored images by a CNN/CycleGAN after L2L training with paired/unpaired images, and the corresponding image of Y113 (B). Scale bar: 20 µm. (C) Training results for two ROIs (6 µm×6 µm). From left to right: input (5H11), the restored images by a CNN after N2N and L2L training with an *L_1_* loss function, the restored image by a CycleGAN, and a 20-frame average. (D) Corresponding benchmark images (Y113) for L2L training and its predictions by a CNN after L2L training as outlined above. A CNN that was trained with L2L data in-paints focal adhesions (see white arrowheads) and reduces cytosolic protein (see ROI number 2) for both the training input and benchmark. Images shown were excluded from the network training.

We also trained a CNN with image pairs of two non-identical labels against the focal adhesion protein paxillin (PXN) (Martino et al., 2018), with the aim to reduce immunofluorescence signal in the images that stems from cytosolic protein. Image pairs of fixed MeT5A (*N*=47), HeLa (*N*=17) and U2OS (*N*=13) cells, dual labelled with the monoclonal anti-PXN antibodies 5H11 and Y113, were acquired. As expected, the raw immunofluorescence images of both antibodies showed correlating focal adhesion structures in the cells but also a diffuse signal throughout the cell body that differed in relative intensity to the focal adhesion signal between the two clones (compare Fig. 3A, left, and Fig. 3B). For all cell lines, we observed the same trend; the images of antibody Y113 showed a higher focal adhesion-to-cytosolic signal ratio than images of 5H11. To determine whether the cytoplasmic signal originated from clone-dependent binding to protein in the cytosol or unspecific binding, short hairpin (sh)RNA-mediated *PXN* knockdown MeT5A cells were generated (see ‘Generation and verification of knockdown PXN MeT5A cells’ in Materials and Methods). The knockdown was confirmed via a qPCR analysis that showed a knockdown efficiency of >70% on mRNA level. Additionally, PXN protein levels were substantially lower in the shRNA PXN cells, as analysed by western blotting (Fig. S6A,B). For both antibodies, the immunofluorescence signal of PXN was reduced in images of fixed shPXN cells in both the focal adhesions and in the cytoplasm (Fig. S6C). Hence, the cytoplasmic signal that is observed in images of both clones is largely not caused by unspecific binding. Instead, the different relative cytosolic content in cell images of 5H11 and Y113 indicates a differing accessibility of the respective binding sites for both clones for protein that is cytosolic or bound to focal adhesions, and that cytosolic PXN likely functions as a readily available replenishable buffer for focal adhesion-localised PXN.

All cell lines were prepared and imaged under the same conditions, and were used indistinguishably to generate the training data (see ‘Training the CNN’ section in Materials and Methods). For the cell images of 5H11/Y113 (*N*=77), mean Michelson contrast values of 0.57±0.07/0.99±0.01, and mean±s.d. RMS contrast values of 0.15±0.01/0.18±0.02 were calculated. Cell images of 5H11/Y113 were used as input/benchmark to train a network to decrease cytosolic signal in images of focal adhesions. To test whether an artificial network can also be trained with unpaired L2L data, a CycleGAN was trained and its performance was compared to the CNN (see ‘Training the CycleGAN’ in Materials and Methods). Further results for other datasets are shown in Fig. S7.

In Fig. 3A, the restorations of a representative training input (5H11) by a CNN (using an *L_1_*) and a CycleGAN are shown after L2L training with aligned and unaligned images, respectively. Both networks learned to reduce cytosolic content in images of 5H11 and selectively recovered focal adhesion structure, but the CNN outperformed the CycleGAN for this task. In the generated image of the CycleGAN, high intensity areas in the cell cytoplasm that did not stem from focal adhesions were, in part, still present in the predicted images with a tendency to translate intensity fluctuations in the input as weak structure (Fig. 3C, bottom). A similar effect was observed after training a CNN with an *L_MS-SSIM_* with N2N data (Fig. S5E). Here, intensity fluctuations in the cytosol were, as observed with the caveolae dataset (see previous section), artificially accentuated in the restorations, which became more pronounced with a small *M*, leading to very different qualitative N2N results after using an *L_1_* and an *L_SSIM_*. These artefacts were much less pronounced in the L2L results (Fig. S5E).

Contrary to the results of the evaluation (Table 1) that, again, indicated the highest network performance after using an *L_5S-SSIM_*, subjectively, training the CNN with an *L_1_* led to the best L2L results overall. Cytosolic signal was significantly lowered in the restorations after L2L training compared to the 20-frame average image or N2N result, and focal adhesions were selectively recovered and appeared with increased contrast (Fig. 3C). Also, the trained CNN in-painted focal adhesion structures that were inhomogeneously labelled (see white arrowheads in Fig. 3C). In addition, we found that the trained CNN reduced cytosolic background signal in the training benchmark (Y113), although these images were not used as input for L2L training (Fig. 3D).

### Training a CNN to separate cellular structures in superposed images

**Fig. 4.**
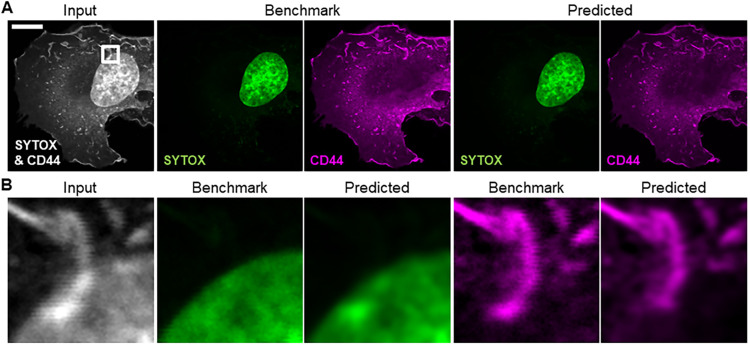
**Qualitative results after training a CNN to separate cellular structures in superposed images of a nuclear stain and an antibody against a plasma membrane protein.** (A) Training input and benchmark images of a MeT5A cell that was dual labelled with the nuclear stain SYTOX Green and an anti-CD44 antibody, and corresponding reconstructions after training a CNN with an L_3S-SSIM_. Scale bar: 10 µm. The image pairs were obtained via sequential imaging by changing the excitation wavelength. (B) Qualitative result for an ROI (5 µm×5 µm). Prediction success is dependent on the level of superposition of both labels. Structures appear slightly blurry in the restorations compared to the benchmark, but image noise and jitter are reduced. The images shown were excluded from the training.

Lastly, the ability of a CNN to transfer style between immunofluorescence images with correlating structural signal was tested by training a network to separate superposed confocal images of two different cellular targets. For that, fixed MeT5A cells were stained with the nuclear marker SYTOX Green and labelled with an antibody against CD44, a plasma membrane protein. Then, image pairs (*N*=58) were acquired of both markers (superposed) and of each marker separately (see the imaging section in Materials and Methods). The spatial distribution in the cell of both targets partly overlapped in the images but the targets were structurally distinguishable, with the nucleus appearing with high intensity in the cell centre and CD44 distributed at the periphery of the cell (Fig. 4A). The CNN was trained with the superposed image of both the nucleus and the plasma membrane as training input in a two channel image, and the separate cell images of SYTOX and CD44, respectively, as training benchmark (see ‘Training the CNN’ in Materials and Methods). The qualitative results in Fig. 4 for an example image pair show that a CNN separated the nucleus and plasma membrane marker in the input image successfully after the training. Noticeably, similar to N2N, image jitter and noise that were present in the input image were removed by the network in the restorations (Fig. 4B). However, the CNN slightly blurred structure in the restorations, and overlapping structures in the nuclear area were mostly, but not fully, recovered in the images of CD44. A loss function-dependent evaluation of the network performance showed no clear trend as to which loss function was best suited to train the CNN as a separator of the two structures (Table 1).

### Testing the robustness of L2L

The robustness of L2L training was evaluated for the actin (*N_tot_*=68), tubulin (*N_tot_*=51), caveolae (*N_tot_*=60) and PXN (*N_tot_*=77) datasets by analysing how much the network performance was impacted by using specific image pairs for the training data generation. To carry this out, repeated cross validations were conducted with randomly selected image pairs *N* from the total dataset (*N_tot_*). For the actin and PXN dataset, we conducted 8-fold cross validations with *N*=8, 16, 32 and 64; for the tubulin and caveolae dataset, we conducted 10-fold cross validations with *N*=10, 30 and 50. We increased the number of repetitions for small *N*, and the epoch number for the training was linearly adapted to *N* to prevent overfitting (see ‘Training the CNN’ in Materials and Methods; Table 3). The difference between this approach and the default validation in the CSBDeep framework is that in the latter the validation is conducted via a train/test split of the training data, which is generated from all raw image pairs. This way, image patches generated from a specific image pair appear in both, the test (validation) and the training data, making it impossible to assess how much the network performance depends on whether a specific image pair is used for the training.

**Fig. 5.**
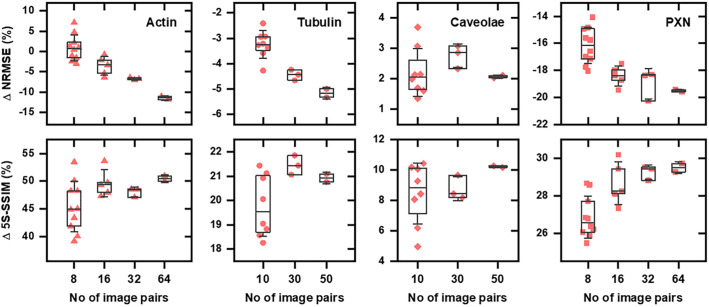
**Repeated cross validation for L2L training*.*** The mean relative change (input/benchmark versus restoration/benchmark) of the NRMSE and 5S-SSIM index after L2L training with image pairs of different cellular structures. Boxes represent 25th and 75th percentiles with median, whiskers represent standard deviations. The image pairs for the trainings were generated of cells that were dual labelled for the actin cytoskeleton (*N_tot_*=68), tubulin (*N_tot_*=51), caveolae (*N_tot_*=60) or PXN (*N_tot_*=77), dependent on the number of raw image pairs that were randomly selected from the total dataset for the cross validation. Each data point is the mean value for an eightfold (actin and PXN) or tenfold (tubulin and caveolae) cross validation that was repeated for small image pair numbers.

The mean relative change of the NRMSE and 5S-SSIM indices between the input and the restored image (both versus the training benchmark) is displayed in Fig. 5. Each data point represents the mean value of a cross validation dependent on *N*. A Gaussian filter (sigma=1.5) was applied to images of caveolae prior to the calculations, as noise levels were so high in the raw input images that no trend was observable for unprocessed data. The results for nearly all datasets showed a similar trend: even when using a small *N*, the structural similarity to the benchmark increased for restored images compared to the input. However, the calculated ΔNRMSE and Δ5S-SSIM were dependent on the particular images that are used for the training – indicated by a wide distribution for small *N*. This could indicate that certain acquired image data are better suited for training the network, or easier to predict for the network. As expected, on average, the highest performance was achieved using a high *N*. Although the results for Δ5S-SSIM indicate that with *N*>30 or 32 the network performance was relatively consistent for all datasets, the ΔNRMSE improved continuously with increasing *N*. Here, the trend of ΔNRMSE deviated for the caveolae dataset for which, altogether, only slight changes were observed between different *N*, without a clear trend.

## DISCUSSION

We show that a CNN can be successfully trained to reduce unspecific label-induced fluorescence signals detected in the cell cytoplasm in immunofluorescence images of cellular structures, requiring images of two non-identical labels of a target for the training that exhibit systematic sample differences. L2L is different to restoration methods that use images of the same label as training data, e.g. N2N. After N2N training a CNN restores all signals originating from the label without distinction, whereas a CNN systematically learns to lower non-structural signals after L2L training. We found that a network trained with L2L data restores images with high contrast of the target structure, even compared to the training benchmark or its high frame average image (see, for example, Fig. 2A,C). Such a CNN further improved the images that were used as the benchmark for the training (Fig. 3D; Fig. S8).

The network performance is dependent on the level of correlation between the images of the two labels and the training loss functions. Using a single-scale SSIM loss function for the training increased the likelihood of hallucination effects in restored images after L2L training, especially for the PXN dataset in which cytosolic content in the cell body was not entirely without structure and present in both training input and output. However, an *L_MS-SSIM_* was better suited for training a network to restore sharp cellular structures. Notably, the restorations increasingly (with *M*) converged to results obtained with an *L_1_* (Fig. S2, Fig. S5). For images of actin, tubulin and caveolae we determined that the best results were obtained after using an *L_3S-SSIM_*. Here, unspecific background signals differed sufficiently between the images of both labels. For the PXN dataset, an *L_1_* was more suitable for training a CNN to recover focal adhesions only, likely due to the correlating cytosolic signal in the training images, albeit both at different relative intensity to the focal adhesion signal (Fig. 3A,B).

Thus far, most deep learning-based image restoration methods have relied on image pairs of the same label that were acquired under different imaging conditions. Here, our N2N results suggest that powerful loss functions, such as the *L_MS-SSIM_*, are only of limited use (e.g. the N2N results in Fig. S5B,E). Using an *L_MS-SSIM_* for N2N training led to a significantly higher occurrence of artefacts compared to L2L, in which cytosolic non-structural content (as present in the training input for the actin, caveolae and PXN datasets) was restored with accentuated artificial contrast. However, the qualitative N2N and L2L results for the caveolae dataset indicate that a CNN picks up structure much more efficiently in very noisy images when using an *L_3S-SSIM_* instead of an *L_1_* for the training (Fig. 2C). But using two STED images of the same label (D1P6W) as training data caused the network to hallucinate significantly when using an *L_MS-SSIM_*, with cytosolic background signals recovered as structure. Also, some N2N results exhibited artefacts that might originate from static image corruptions introduced by the imaging system itself, which then would be present in both noise realisations of a sample (Fig. 2C; Fig. S5B). A clear improvement between the restored images after N2N training with an *L_1_* and an *L_3S-SSIM_*, respectively, is only observed for the tubulin dataset, in which the background signal appears very unspecific in images of the tubulin marker (DM1A) (Fig. 2A).

Although clean benchmark data are not required for L2L and N2N training, in images of structures that are not sufficiently resolved by the imaging technique, either *a posteriori* knowledge about the structure or clean reference data are required to assess the qualitative performance of a trained CNN. This was especially noticeable in cell images of tubulin, in which the fine microtubule network is not fully resolved with confocal microscopy (Fig. 2A,B). We observed erroneous predictions by a CNN in image regions where the microtubule network was densely packed after both N2N and L2L training. Further, the sharpening of structure with L2L after using an MS-SSIM loss function for the training – although advantageous for images of tubulin (Fig. S4C) for which we know that the true microtubule diameter is not resolved with confocal microscopy – may be less desirable in images of other cell structures.

We found that metrics such as the PSNR and NRMSE are inadequate to forecast which loss function yields the best restorations by a CNN after L2L training, and this is exacerbated by the unavailability of clean reference images of the respective target structure. For instance, the calculated metrics for the L2L results for all datasets barely deviated for different *L_MS-SSIM_*, although, qualitatively, differences in the restorations were observed (Table 1). Additionally, although the calculated SSIM indices indicate scarcely any correlation between the input and benchmark, with values around 0.05-0.15 for all L2L datasets, the correlation was higher according to the 5S-SSIM indices (by a factor of ∼3). Therefore, a high-scale SSIM index might be more suitable for detecting correlation in fluorescence images of cellular structure than other metrics, likely because microscopy images are the convolved signal of a sample volume rather than the strict two-dimensional depiction of a sample at a specific section. This observation fits with the MS-SSIM index theory (Wang et al., 2003).

As expected, the evaluation of repeated eightfold/tenfold cross validations (dependent on the number of raw image pairs that were used to generate the training data) showed that using a high number of image pairs to train a CNN for the style transfer between two labels is advisable (Fig. 5). The mean PSNR and 5S-SSIM index significantly increased even after L2L training with a small number of image pairs, but the results were dependent on the selected image pairs. Although the evaluation would be more meaningful if each cross validation had been conducted with unique image pairs instead of image pairs but that were randomly selected from a finite dataset, the results indicate that a network converges to the optimal result of a particular dataset during training (Fig. 5).

Initially, it was unclear whether the difference in the training data would increase hallucinations by a CNN after L2L training. For instance, in the caveolae dataset, the sample differences between input and output were quite significant (compare Fig. S5A,C), and inhomogeneous labelling of the microtubule structure by the anti-tubulin antibody YOL1/34 (benchmark) was not visible in the training input (DM1A) (compare Fig. S2D,F). Yet, we could not observe that the CNN introduced artificial structure in the restored images after L2L training that resembled either trend. Instead, our results suggest that sample differences can be advantageous when training with an *L_MS-SSIM_* to lower the occurrence of hallucination effects observed after N2N training. However, an in-painting effect was observed for L2L, primarily in restorations of AC-15 (actin; Fig. 1C,D) and 5H11 (PXN; see Fig. 3C,D). Consequently, L2L can be used to correct images for inhomogeneous binding of a stain or antibody but restorations cannot be used to quantify the distribution of a target protein in a cell. Instead, L2L could serve as an image pre-processing step to extract the binary information about the location of a structure in the cell (see examples in Fig. S8). Notably, the systematic recovery of specific structure and the adaptability of L2L to images of a multitude of targets potentially makes L2L superior to classical image processing methods (as shown in Fig. S3 and Table S1).

Training data for L2L can be generated with one imaging setup using two detectors simultaneously, which makes the images independent of stage drift and sample dynamics, and is time efficient. However, contrary to N2N, L2L requires sample preparation with two markers that exhibit systematic differences in the respective images to allow training for a useful style transfer between labels. Therefore, not all label pairs of a target structure are suitable for generating the necessary training data. We found that the calculated RMS and Michelson contrast values for images of two labels were good indicators for assigning labels to ‘input’ and ‘benchmark’. Here, training a CNN with the reverse order or pairing the labels in both directions resulted in either worse or comparable prediction success.

We also trained a CycleGAN with unaligned label pairs of a target structure (Fig. 3A,C; Fig. S7). Although the generated images of a trained CycleGAN exhibited reduced unspecific cytosolic signals, it was outperformed by a trained CNN. As the generator in the CycleGAN is trained to fool a discriminator based on noisy benchmarks, either little to no change to the input image was observed (tubulin/caveolae) or artefacts were introduced (actin/PXN) by the network to match the style of the reference image. Prior denoising of the images via Gaussian filtering led to slightly better results (Fig. S7). A higher performance might be achieved with cleaner reference images.

The ability of a CNN to selectively restore specific cell structure is also highlighted in this work by training a CNN to separate a nuclear marker and a plasma membrane protein label in superposed immunofluorescence images (Fig. 4). Our results show that CNNs could be used in the future to separate the fluorescence signals from multiple markers in microscopy images that were acquired with imaging setups that have a limited number of excitation sources or detectors. L2L could also be applied in multiplex imaging experiments if antibodies are not selected based on performance but compatibility issues between the species in which they are raised. Moreover, L2L could be considered for post-processing in live-cell imaging, in which high-performance labels are rare. Training data can be generated post image acquisition *in vitro* by fixing the cells and labelling with a higher performing antibody against the target structure. Our results for the caveolae dataset suggest that training a CNN with L2L data might be particularly advantageous to restore noisy images, as it allows the implementation of an MS-SSIM loss function without introducing artefacts that we otherwise observed after training with images of the same label (Fig. 2C; Fig. S5B). Here, L2L results exhibit a high structure-to-background signal ratio, clearly outperforming high-frame average images that were acquired with the STED microscope.

In conclusion, we present a new deep learning-based image restoration method for images of cellular structures that utilises the varying performance of labels in immunofluorescence microscopy; we call this method L2L. With L2L, we show that by training a CNN for a style transfer between two non-identical labels of a shared target, the network can be systematically trained to reduce unspecific cytosolic background signals and enhance structural contrast in immunofluorescence images. Like other methods, L2L relies on the convention of the network to underestimate inherently unpredictable signal. However, with L2L, not only image noise but also label-induced fluorescence signals in the cell specimen can be reduced in the images after selecting appropriate training data. The ability to significantly lower unspecific binding, inhomogeneous labelling of a structure or binding to cytosolic protein in immunofluorescence images makes L2L, to our knowledge, unique in comparison to other deep learning-based image restoration methods that are currently used in cell biology.

## MATERIALS AND METHODS

### Cell culture

For imaging, the human mesothelial cell line MeT5A (ATCC CRL-9444), the adenocarcinoma cervical cancer cell line HeLa and the human osteosarcoma cell line U2OS were used. HeLa cells were a gift from Margaret Cunningham and U2OS cells were a gift from Kathryn McIntosh (both Strathclyde Institute of Pharmacy and Biomedical Sciences, Glasgow, UK). MeT5A cells were cultured in RPMI-1640 medium (Corning), supplemented with 10% (v/v) fetal bovine serum (FBS) (Labtech), 100 μg/ml penicillin-streptomycin (Gibco), 1 mM sodium pyruvate (Gibco), 2 mM L-glutamine (Gibco) and 2 mM HEPES buffer solution (Gibco). HeLa and U2OS cells were cultured in Dulbecco's modified Eagle medium (DMEM) plus GlutaMAX medium (Gibco) supplemented with 10% (v/v) FBS and 100 μg/ml penicillin-streptomycin. Human embryonic kidney cells HEK293T were grown in DMEM supplemented with 100 μg/ml penicillin-streptomycin and 2 mM L-glutamine. All cells were kept at 37°C/5% CO_2_ in a humidified atmosphere.

### Generation and verification of knockdown paxillin MeT5A cells

Knockdown of MeT5A cells was achieved by a shRNA-mediated knockdown using pKLO.1 vectors coding for shRNAs targeting paxillin (TRC N0000123137) or control. HEK293T cells were used for virus generation, and the virus was harvested, filtered and added to polybrene-treated MeT5A cells. The retroviral transduction of MeT5A cells was followed by puromycin selection. Stable knockdown cell lines were verified by qPCR (mRNA abundance) and western blots (protein abundance). For western blotting, cell lysates were prepared in reducing lysis buffer, boiled for 10 min and separated on conventional homemade SDS gels, and developed onto Medical Blue Sensitive X-Ray Film (Scientific Laboratory Supplies). The PXN specific antibody Y113 was used (rabbit; ab32084, Abcam). Ponceau staining of the PVDF membrane was carried out to establish equal loading. For qPCR (RT-qPCR), mRNA was extracted from cells using an RNeasy Plus Mini kit (Qiagen). cDNA was generated using a High-Capacity cDNA Reverse Transcriptase kit (Applied Biosystems). qPCR was performed on 1 ng of cDNA using Brilliant III Ultra-Fast SYBR Green QPCR Master Mix (Agilent Technologies) and an Applied Biosystems QuantStudio 5 Real-Time PCR System. Expression of paxillin was analysed and normalized to hypoxanthine-guanine phosphoribosyltransferase (*HPRT1*) levels. qPCR data originate from five independent biological replicates, plotted in Prism (GraphPad) and analysed using Mann–Whitney.

### Sample preparation for immunofluorescence microscopy

If not stated otherwise, cells were plated onto #1.5 coverslips a day prior to fixation, then fixed with 4% paraformaldehyde for 15 min at 37°C, followed by a permeabilisation step with 2.5% FBS and 0.3% Triton X-100 in PBS for 30 min at room temperature (Rausch and Hansen, 2019). HeLa cells that were labelled for actin were fixed 6 h after plating. The following antibodies or stains were used to generate cell specimens dual labelled for the same target structure: monoclonal anti-β-actin antibody AC-15 conjugated to Alexa Fluor 488 (1:250; mouse; ab6277, Abcam) and the phalloidin-Atto 565 stain (1:100; 94072, Sigma-Aldrich) to visualise the actin cytoskeleton in MeT5As; monoclonal antibodies YOL1/34 conjugated to Alexa Fluor 488 (1:500; rat; ab195883, Abcam) and DM1A (1:250; mouse; T6199, Sigma-Aldrich) for α-tubulin labelling in MeT5As; monoclonal antibodies 4H312 (CAV1) (1:200; mouse; sc-70516, Santa Cruz Biotechnology) and D1P6W (CAVIN-1) (1:200; rabbit; 69036, Cell Signaling Technology) for labelling caveolae in MeT5As; and monoclonal antibodies Y113 (1:250; rabbit; ab32084, Abcam) and 5H11 (1:500; mouse; MA5-13356, Life Technologies) for PXN labelling in MeT5A, U2OS and HeLa cells. Further, fixed MeT5A cells were labelled with a nuclear SYTOX Green marker (S7020, Life Technologies) and a monoclonal anti-CD44 antibody (1:100; rat; MA4400, Life Technologies). The following secondary antibodies were used: anti-rabbit IgG Alexa Fluor 488 (1:200; donkey; A-21206, Life Technologies) to conjugate Y113; anti-rat IgG Alexa Fluor 555 (1:100; goat; A-21434, Life Technologies) to conjugate the antibody against CD44; anti-mouse IgG Alexa Fluor 594 (1:200; goat; A32742, Life Technologies) to conjugate 4H312; anti-mouse IgG Alexa Fluor 633 (1:200; goat; A-11001, Life Technologies) to conjugate DM1A and 5H11; and anti-rabbit IgG Atto 647N (1:200; goat; 40839, Sigma-Aldrich) to conjugate D1P6W. All immunofluorescence samples were mounted in ProLong Glass Antifade Mountant (Life Technologies) onto microscope slides.

### Imaging

#### Confocal microscopy

Image pairs of the immunofluorescence sample labelled for the actin cytoskeleton, tubulin and PXN, as well as SYTOX Green and CD44, were taken using a commercial confocal laser scanning microscope (Leica TCS SP8, Leica Microsystems), using a HC PL Apo 63×/1.4 N.A. CS2 objective with no digital zoom (for image sizes see Table 2). To acquire data for L2L training, both markers in the individual sample were imaged simultaneously using the two in-built photomultiplier detectors, each equipped with a prism-based tunable spectral filter. Each field of view was imaged twice to acquire the two noise realisations of the samples that were used for N2N training of a CNN. The sample dual labelled for actin was excited with a 488 nm and 552 nm laser line, and the two spectral detectors were set to detect light between 495-540 and 560-700 nm. The samples dual labelled for tubulin and PXN were excited with a 488 nm and 638 nm laser line, and the two spectral detectors were set to detect light between 495-620 nm and 645-750 nm. The immunofluorescence sample labelled for SYTOX Green and CD44 was imaged by setting the spectral range of the detector to 560-660 nm. Separate images of SYTOX and CD44 were acquired by exciting the sample with a 488 and 552 nm laser line, respectively. A corresponding superposed image of both markers was acquired by exciting the sample with both lasers simultaneously.

**Table 2.**
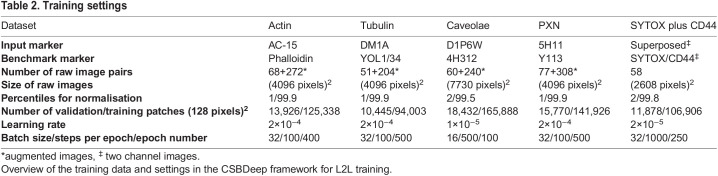
Training settings

#### Stimulated emission depletion microscopy

Fixed MeT5A cells dual labelled for tubulin were further imaged with a Leica SP8 TCS 3X STED microscope, using a Leica HC PL APO 100×/1.40 Oil STED WHITE objective (15506378, Leica). The sample was excited with a supercontinuum white light laser at 488 nm and 633 nm for confocal imaging, and at 633 nm for STED imaging. For confocal imaging, two Leica photomultiplier tube detectors were set to detect light between 498-600 nm and 643-743 nm. For STED imaging, a Leica HyD hybrid detector was set to detect light between 643-743 nm. STED depletion was performed using a Leica 775 nm depletion laser set to 50% with time gating from 0.3-8 ns. Pairs of confocal and STED images were acquired with a 15 nm pixel size. Sequential STED image pairs of the cell specimen labelled for caveolae were acquired by first exciting the sample at 646 nm and detecting light between 656-750 nm, then exciting at 591 nm and using a spectral range of 601-650 nm for the detection. The depletion laser power was set to 100% with time gating from 0.3-8 ns, and the pixel size was set to 10 nm.

### Image drift correction

Fluorescence images that were taken sequentially were corrected for stage drift by estimating the extent of the displacement between the images. A total of 100 image pairs of size 60×60 pixels were generated for each image in a temporal image stack, after applying a Gaussian filter (sigma=2) and using the first detected image as benchmark. For that, the create_patches function from the CSBDeep framework was used, which cuts random image patch pairs in non-background areas of the image. For each created image patch, the SSIM index (filter size=11, sigma=1.5) was calculated by shuffling the uncorrected image patch relative to the benchmark patch by 5 pixels in all directions, effectively cutting both into 50×50-pixel patches. The shuffling vector that, on average, yielded the highest structural similarity between both images was selected as optimal position for the sequential image, and the images were cropped accordingly.

### Data augmentation and pre-processing

Images were augmented to create more training data. To achieve this, the images were interpolated, using the zooming factors 0.5, 0.75, 1.25 and 1.5 (0.8, 0.9, 1.1 and 1.2 for the caveolae dataset), and then randomly rotated by 0°, 90°, 180° or 270°. STED images for the caveolae dataset were pre-processed by adding Poisson noise to broaden the image histogram that, due to being acquired with hybrid detectors, was nearly binary in the raw images, resulting in poor results for the network training. Prior to the training patch generation in the CSBDeep framework, a percentile normalisation was conducted (Table 2).

### Training the CNN

For N2N and L2L training, the CSBDeep framework was used, which is a CNN with U-Net architecture (Ronneberger et al., 2015) that was developed for CARE in fluorescence microscopy (Weigert et al., 2018). The CSBDeep framework (version 0.6.0) was downloaded from GitHub and used with its default settings unless stated otherwise (https://github.com/CSBDeep/CSBDeep).

In the CSBDeep framework, training patches were generated randomly from the raw images after pre-processing (see previous section). Half of the training patches were generated from the raw images, and the other half from augmented images. Validation data were generated via a 90/10 train/test split, and the training and validation loss were monitored to rule out overfitting.

A least absolute deviation loss function (*L_1_*) and different multiscale SSIM loss functions were used for the training [*L_SSIM_* (*M*=1), *L_3S-SSIM_* (*M*=3), *L_5S-SSIM_* (*M*=5)]. For an *L*_*3S-SSIM*_, the weights were set to 0.2096, 0.4659 and 0.3245, and for a *L*_*5S-SSIM*_, the size for the Gaussian filter was set to 7, which is the maximum possible filter size for the selected patch size; otherwise the suggested settings by Wang et al. (2003) were used.

The settings that were applied for the L2L training are shown in Table 2. The same settings were used to train the network for N2N, but the training data were generated from two sequential images of the respective antibody. To train the CNN as a separator of two cellular markers (the SYTOX Green stain and a CD44 antibody) in superposed immunofluorescence images, the CSBDeep Framework was trained with two channel images, where the input consisted of the superposed image in both channels, and the separately acquired images of SYTOX and CD44 as the output channels (see Table 2, right).

To further evaluate L2L, repeated eightfold or tenfold cross validations were conducted on the datasets using the settings outlined in Table 3. The selection of the raw image pairs from the total dataset for each cross validation and the fold allocation were conducted randomly in Python. The image pairs for each training were generated as described above, including the pre-processing, and disabling the train/test split in the CSBDeep Framework. The relative change of the NRMSE and 5S-SSIM indices were calculated between input/benchmark and prediction/benchmark for the test images of each fold, deriving an average for each cross validation (Fig. 5).

**Table 3.**
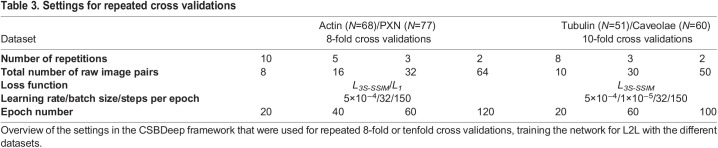
Settings for repeated cross validations

### Training the CycleGAN

The implementation of a CycleGAN in Pytorch was downloaded from GitHub and, if not stated otherwise, trained with the default parameters (https://github.com/junyanz/pytorch-CycleGAN-and-pix2pix) (Zhu et al., 2017). The CycleGAN was trained with unaligned images of the actin, tubulin, caveolae and PXN datasets that were pre-processed as outlined in the previous sections (see also Table 2), using a least squares GAN with a ResNet-9 generator architecture and a 70×70 PatchGAN discriminator architecture. Training was conducted with a batch size of 4, an epoch number of 4/10 (3/9 with linear decay of the learning rate) for the PXN/other dataset(s) and a scaling factor of 0.0005 for the network initialization.

### Image processing and analysis

Image processing and analysis was conducted in Python using the following functions/libraries in default, if not stated otherwise. To compare L2L with classical image processing methods (Fig. S3, Table S1), the following steps were undertaken for images of actin/tubulin/caveolae/PXN: Gaussian filters were applied with a sigma of 2/2/3/2 using ndimage.gaussian_filter in scipy (Virtanen et al., 2020); for rolling-ball background subtraction, subtract_background_rolling_ball from https://github.com/mbalatsko/opencv-rolling-ball was used with a radius of 20/10/5/5; top-hat filters were applied with a filter size of 11/25/13/17; and contrast limited adaptive histogram equalization (CLAHE) was conducted with a grid size of 11/7/7/7, using getStructuringElement(cv2.MORPH_RECT) or createCLAHE, respectively, from OpenCV (Bradski, 2000).

The FWHM was derived from 20 randomly selected line profiles across single microtubules in images of tubulin, by averaging the line profile across 20 pixels and determining the Gaussian fit with scipy (Virtanen et al., 2020) (Fig. S4C).

To generate distance maps or binarised images (Fig. S8), the following pre-processing steps were undertaken using above mentioned functions: for images of actin, a rolling-ball background subtraction (radius=10), a top-hat filter (filter size=7) and CLAHE (tile size 11) were applied; for images of tubulin, a rolling-ball background subtraction (radius=10) and a top-hat filter (filter size=11) were applied; for images of caveolae, a Gaussian filter (sigma=0.75) and a rolling-ball background subtraction (radius=5) were applied; and for images of PXN, a rolling-ball background subtraction (radius=5) was applied. Lastly, objects below a size of 20 pixels (caveolae)/50 pixels (all else) were removed. Binary images were generated using the 75th/60th/93th/90th percentile as threshold for images of actin/tubulin/caveolae/PXN. Distance maps were generated using scipy (Virtanen et al., 2020).

## Supplementary Material

Supplementary information

Reviewer comments

## References

[JCS258994C1] Bates, M., Huang, B., Dempsey, G. T. and Zhuang, X. (2007). Multicolor super-resolution imaging with photo-switchable fluorescent probes. *Science* 317, 1749-1753. 10.1126/science.1146598 17702910 PMC2633025

[JCS258994C2] Belthangady, C. and Royer, L. A. (2019). Applications, promises, and pitfalls of deep learning for fluorescence image reconstruction. *Nat. Methods* 16, 1215-1225. 10.1038/s41592-019-0458-z 31285623

[JCS258994C3] Bradski, G. (2000). The OpenCV library. *Dr. Dobb's J. Software Tools* 120, 122-125.

[JCS258994C4] Caicedo, J. C., Roth, J., Goodman, A., Becker, T., Karhohs, K. W., Broisin, M., Molnar, C., Mcquin, C., Singh, S., Theis, F. J. et al. (2019). Evaluation of deep learning strategies for nucleus segmentation in fluorescence images. *Cytometry Part A* 95, 952-965. 10.1002/cyto.a.23863 PMC6771982 31313519

[JCS258994C5] Christiansen, E. M., Yang, S. J., Ando, D. M., Javaherian, A., Skibinski, G., Lipnick, S., Mount, E., O'Neil, A., Shah, K., Lee, A. K. et al. (2018). In silico labeling: predicting fluorescent labels in unlabeled images. *Cell* 173, 792-803. 10.1016/j.cell.2018.03.040 29656897 PMC6309178

[JCS258994C6] Dugina, V., Zwaenepoel, I., Gabbiani, G., Cleì Ment, S. and Chaponnier, C. (2009). Beta and gamma-cytoplasmic actins display distinct distribution and functional diversity. *J. Cell Sci.* 122, 2980-2988. 10.1242/jcs.041970 19638415

[JCS258994C7] Falk, T., Mai, D., Bensch, R., Çiçek, Ö., Abdulkadir, A., Marrakchi, Y., Böhm, A., Deubner, J., Jäckel, Z., Seiwald, K. et al. (2019). U-Net: deep learning for cell counting, detection, and morphometry. *Nat. Methods* 16, 67-70. 10.1038/s41592-018-0261-2 30559429

[JCS258994C8] Goodfellow, I. J., Pouget-Abadie, J., Mirza, M., Xu, B., Warde-Farley, D., Ozair, S., Courville, A. and Bengio, Y. (2014). Generative adversarial nets. In *Advances in Neural Information Processing Systems*, pp. 2672-2680.

[JCS258994C9] Hansen, C. G. and Nichols, B. J. (2010). Exploring the caves: cavins, caveolins and caveolae. *Trends Cell Biol.* 20, 177-186. 10.1016/j.tcb.2010.01.005 20153650

[JCS258994C10] Ilangumaran, S., Borisch, B. and Hoessli, D. C. (2010). Signal transduction via CD44: role of plasma membrane microdomains. *Leuk. Lymphoma.* 35, 455-469. 10.1080/10428199909169610 10609783

[JCS258994C11] Jing, Y., Yang, Y., Feng, Z., Ye, J., Yu, Y. and Song, M. (2020). Neural style transfer: a review. *IEEE Trans. Vis. Comput. Graph* 26, 3365-3385. 10.1109/TVCG.2019.2921336 31180860

[JCS258994C12] Khater, I. M., Meng, F., Wong, T. H., Nabi, I. R. and Hamarneh, G. (2018). Super resolution network analysis defines the molecular architecture of caveolae and caveolin-1 scaffolds. *Sci. Rep.* 8, 1-15. 10.1038/s41598-018-27216-4 29899348 PMC5998020

[JCS258994C13] Krull, A., Buchholz, T.-O. and Jug, F. (2019). Noise2Void - Learning Denoising from Single Noisy Images. Proceedings of the IEEE Conference on Computer Vision and Pattern Recognition, pp. 2129-2137.

[JCS258994C14] Larsen, A. B. L., Sønderby, S. K., Larochelle, H. and Winther, O. (2016). Autoencoding beyond pixels using a learned similarity metric. Proceedings of The 33rd International Conference on Machine Learning, vol. 48, 1558-1566. https://proceedings.mlr.press/v48/larsen16.html

[JCS258994C15] Lehtinen, J., Munkberg, J., Hasselgren, J., Laine, S., Karras, T., Aittala, M. and Aila, T. (2018). Noise2Noise: Learning Image Restoration without Clean Data. Proceedings of the 35th International Conference on Machine Learning 80, 2965-2974.

[JCS258994C16] Lim, S., park, H., Lee, S.-E., Chang, S., Sim, B. and Ye, J. C. (2020). CycleGAN with a blur kernel for deconvolution microscopy: optimal transport geometry. *IEEE Trans. Comput. Imaging* 6, 1127-1138. 10.1109/TCI.2020.3006735

[JCS258994C17] Martino, F., Perestrelo, A. R., Vinarský, V., Pagliari, S. and Forte, G. (2018). Cellular mechanotransduction: from tension to function. *Front. Physiol.* 9, 824. 10.3389/fphys.2018.00824 30026699 PMC6041413

[JCS258994C18] Miller, D. M. and Shakes, D. C. (1995). Immunofluorescence microscopy. *Methods Cell Biol.* 48, 365-394. 10.1016/S0091-679X(08)61396-5 8531735

[JCS258994C19] Nogales, E. (2000). Structural insights into microtubule function. *Annu. Rev. Biochem.* 69, 277-302. 10.1146/annurev.biochem.69.1.277 10966460

[JCS258994C20] Ounkomol, C., Seshamani, S., Maleckar, M. M., Collman, F. and Johnson, G. R. (2018). Label-free prediction of three-dimensional fluorescence images from transmitted-light microscopy. *Nat. Methods* 15, 917-920. 10.1038/s41592-018-0111-2 30224672 PMC6212323

[JCS258994C21] Peli, E. (1990). Contrast in complex images. *J. Opt. Soc. Am. A* 7, 2032-2040. 10.1364/JOSAA.7.002032 2231113

[JCS258994C22] Pellegrini, F. and Budman, D. R. (2005). Review: tubulin function, action of antitubulin drugs, and new drug development. *Cancer Investig.* 23, 264-273. 10.1081/CNV-200055970 15948296

[JCS258994C23] Rausch, V. and Hansen, C. G. (2019). Immunofluorescence study of endogenous YAP in mammalian cells. *Methods Mol. Biol.* 1893, 97-106. 10.1007/978-1-4939-8910-2_8 30565128

[JCS258994C24] Rausch, V. and Hansen, C. G. (2020). The hippo pathway, YAP/TAZ, and the plasma membrane. *Trends Cell Biol.* 30, 32-48. 10.1016/j.tcb.2019.10.005 31806419

[JCS258994C25] Ronneberger, O., Fischer, P. and Brox, T. (2015). U-Net: convolutional networks for biomedical image segmentation. *Medical Image Computing and Computer-Assisted Intervention – MICCAI 2015. MICCAI 2015. Lecture Notes in Computer Science* 9351, 234-241. 10.1007/978-3-319-24574-4_28

[JCS258994C26] Saper, C. B. (2009). A guide to the perplexed on the specificity of antibodies. *J. Histochem. Cytochem.* 57, 1-5. 10.1369/jhc.2008.952770 18854594 PMC2605712

[JCS258994C27] Suarez, C. and Kovar, D. R. (2016). Internetwork competition for monomers governs actin cytoskeleton organization. *Nat. Rev. Mol. Cell Biol.* 17, 799-810. 10.1038/nrm.2016.106 27625321 PMC5125073

[JCS258994C28] Suzuki, T., Matsuzaki, T., Hagiwara, H., Aoki, T. and Takata, K. (2007). Recent advances in fluorescent labeling techniques for fluorescence microscopy. *Acta Histochem. Cytochem.* 40, 131-139. 10.1267/ahc.07023 18224244 PMC2156041

[JCS258994C29] Virtanen, P., Gommers, R., Oliphant, T. E., Haberland, M., Reddy, T., Cournapeau, D., Burovski, E., Peterson, P., Weckesser, W., Bright, J. et al. (2020). SciPy 1.0: fundamental algorithms for scientific computing in Python. *Nat. Methods* 17, 261-272. 10.1038/s41592-019-0686-2 32015543 PMC7056644

[JCS258994C30] Von Chamier, L., Laine, R. F., Jukkala, J., Spahn, C., Krentzel, D., Nehme, E., Lerche, M., Hernández-Pérez, S., Mattila, P. K., Karinou, E. et al. (2021). Democratising deep learning for microscopy with ZeroCostDL4Mic. *Nat. Commun.* 12, 2276. 10.1038/s41467-021-22518-0 33859193 PMC8050272

[JCS258994C31] Wagner, C., Singer, D., Ueberschär, O., Stangner, T., Gutsche, C., Hoffmann, R. and Kremer, F. (2011). Dynamic force spectroscopy on the binding of monoclonal antibodies and tau peptides. *Soft Mat.* 7, 4370-4378. 10.1039/c0sm01414a

[JCS258994C32] Wang, Z., Bovik, A. C., Sheikh, H. R. and Simoncelli, E. P. (2004). Image quality assessment: from error visibility to structural similarity. *IEEE Trans. Image Process.* 13, 600-612. 10.1109/TIP.2003.819861 15376593

[JCS258994C33] Wang, H., Rivenson, Y., Jin, Y., Wei, Z., Gao, R., Günaydin, H., Bentolila, L. A., Kural, C. and Ozcan, A. (2019). Deep learning enables cross-modality super-resolution in fluorescence microscopy. *Nat. Methods* 16, 103-110. 10.1038/s41592-018-0239-0 30559434 PMC7276094

[JCS258994C34] Wang, Z., Simoncelli, E. P. and Bovik, A. C. (2003). Multi-scale structural similarity for image quality assessment. *Conference Record of the Asilomar Conference on Signals, Systems and Computers* 2, 1398-1402.

[JCS258994C35] Weigert, M., Schmidt, U., Boothe, T., Müller, A., Dibrov, A., Jain, A., Wilhelm, B., Schmidt, D., Broaddus, C., Culley, S. et al. (2018). Content-aware image restoration: pushing the limits of fluorescence microscopy. *Nat. Methods* 15, 1090-1097. 10.1038/s41592-018-0216-7 30478326

[JCS258994C36] Zhao, H., Gallo, O., Frosio, I. and Kautz, J. (2016). Loss functions for image restoration with neural networks. *IEEE Trans. Comput. Imaging* 3, 47-57. 10.1109/TCI.2016.2644865

[JCS258994C37] Zhu, J. Y., Park, T., Isola, P. and Efros, A. A. (2017). Unpaired image-to-image translation using cycle-consistent adversarial networks. In Proceedings of the IEEE International Conference on Computer Vision. Institute of Electrical and Electronics Engineers Inc., pp. 2242-2251.

