# Supplementary material for: Label2label: training a neural network to selectively restore cellular structures in fluorescence microscopy
Source: J Cell Sci. 2022 Feb 10;135(3):jcs258994. doi: 10.1242/jcs.258994 (PMC8918818; doi:10.1242/jcs.258994)
Supplement: Supplementary information [file joces-135-258994-s1.pdf]

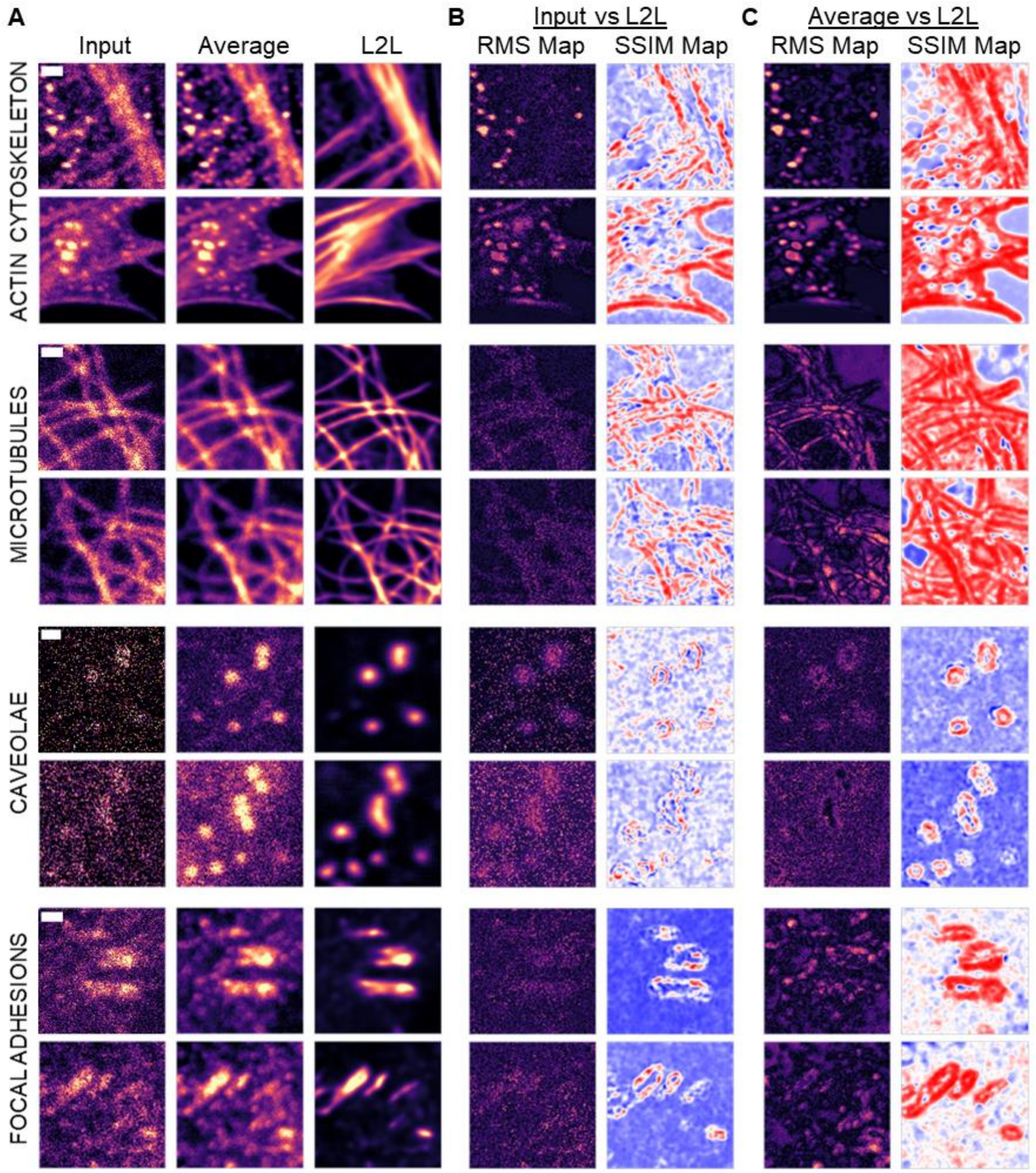

Fig. S1. RMS and SSIM maps between the input and predicted images after L2L training. (**A**) Two representative inputs for L2L training, corresponding 20-frame average images and L2L results for four different cellular structures (*top-to-bottom*): AC-15 (actin), DM1A (α-tubulin), D1P6W (CAVIN-1, an essential caveolae component), and 5H11 (PXN, an essential focal adhesion component). The CNN was trained with a *L1* (PXN)/*L3S-SSIM* (all else) loss function. RMS and SSIM maps between (**B**) the input and predicted images, and (**C**) the 20-frame average images and the predicted images. The LUT of the SSIM maps is selected such that 'blue', 'white' and 'red' indicate a negative, no and a positive correlation between the images, respectively. The trained network reduces cytosolic background signals in the images (RMS maps) and sharpens the cellular structure (SSIM maps). The shown image pairs were excluded from the trainings (scale bar (*top-to-bottom*) = 1/1/0.2/1 µm).

Journal of Cell Science • Supplementary information

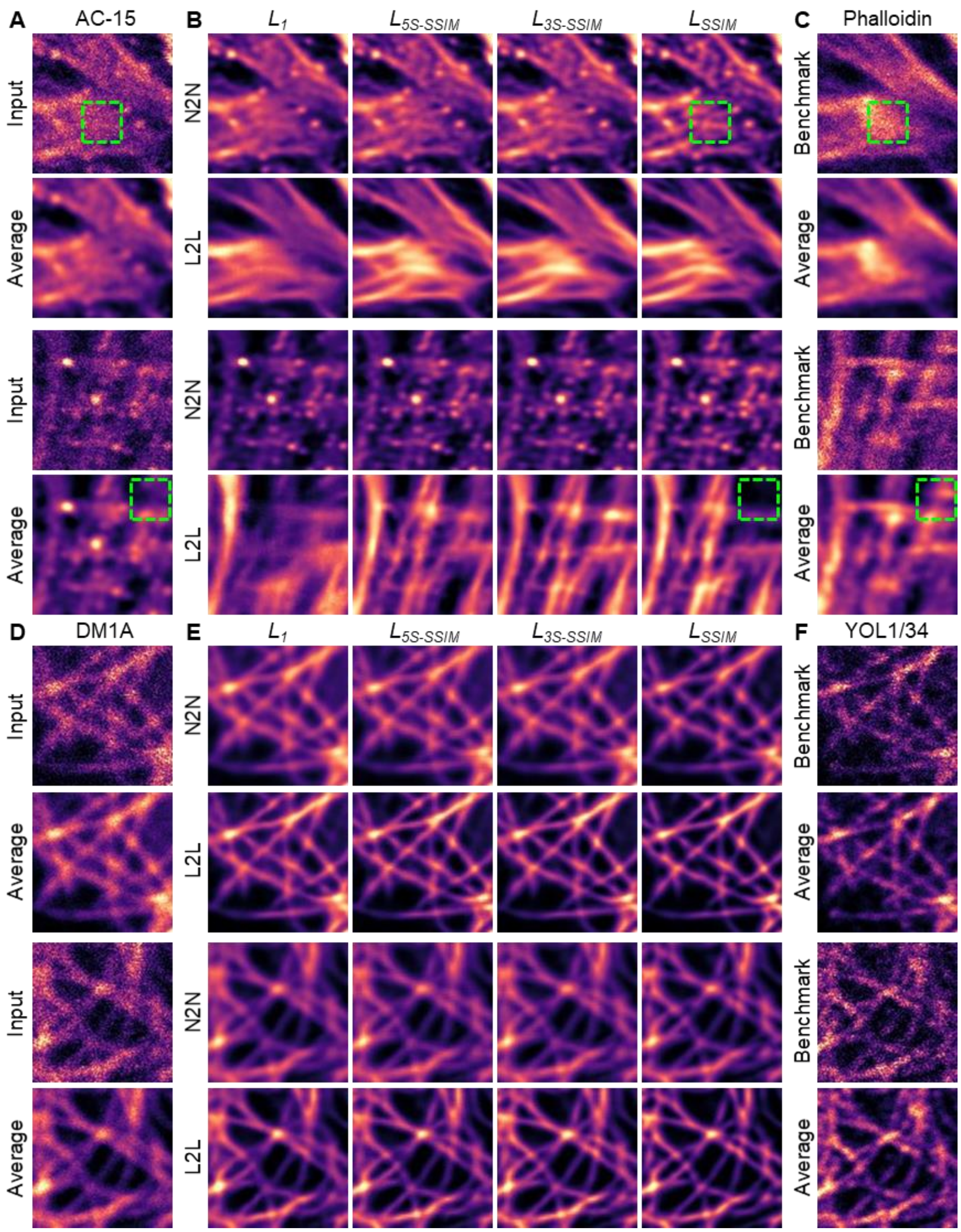

Fig. S2. Loss function-dependent label2label and noise2noise results for confocal images of the actin cytoskeleton and the microtubule network.

(*Left*) Training inputs and corresponding 20-frame average images of (**A**) HeLa cells labelled with AC-15 (actin) and (**D**) MeT5A cells labelled with DM1A (tubulin). (**B**,**E**) Corresponding restorations by a CNN after L2L and N2N training with different loss functions. (**B**) Examples of hallucination effects are highlighted in green boxes. (*Right*) The benchmarks for L2L training and 20-frame average images for the (**C**) actin (phalloidin) or (**F**) tubulin (YOL1/34) dataset, respectively. The shown image pairs were excluded from the trainings, and contrast adjusted to the 2nd and 99.8th percentile (image dimensions: 4.5 µm x 4.5 µm).

Journal of Cell Science • Supplementary information

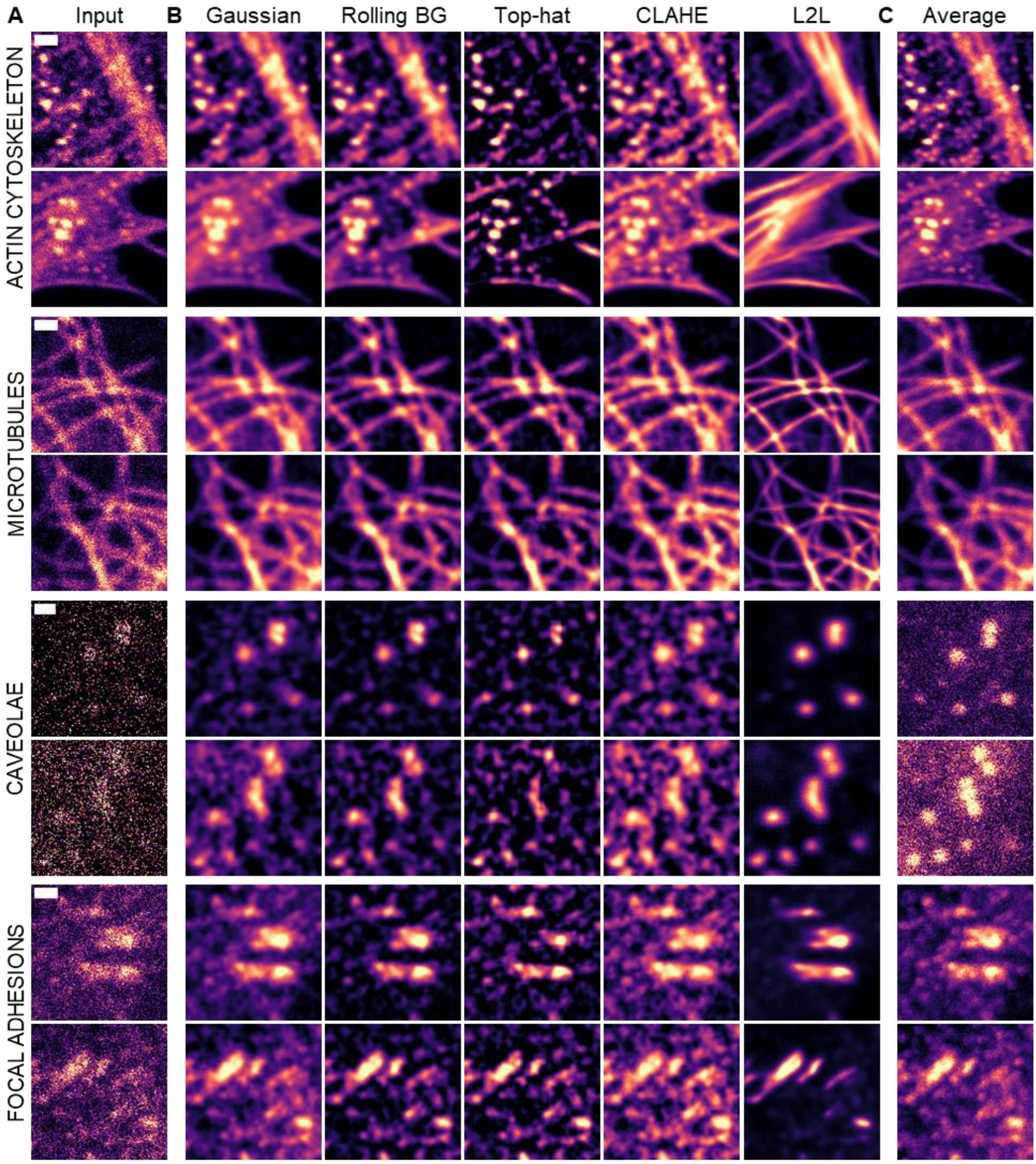

Fig. S3. Classical methods to enhance image contrast versus label2label.

(**A**) Two representative inputs for L2L training for four different cellular structures (*top-to-bottom*): AC-15 (actin), DM1A (α-tubulin), D1P6W (CAVIN-1, an essential caveolae component), and 5H11 (PXN, an essential focal adhesion component). (**B**) Images after applying a Gaussian filter, after applying a Gaussian filter and rolling-ball background subtraction, a Gaussian filter and top-hat filter, a Gaussian filter and contrast limited adaptive histogram equalization (CLAHE), and the predicted images of a CNN after L2L training with a *L1* (PXN)/*L3S-SSIM* (all else) loss function. (**C**) Corresponding 20-frame average images. The shown images were excluded from the network trainings (scale bar (*top-to-bottom*) = 1/1/0.2/1 µm). For a quantitative evaluation see **Table S1**.

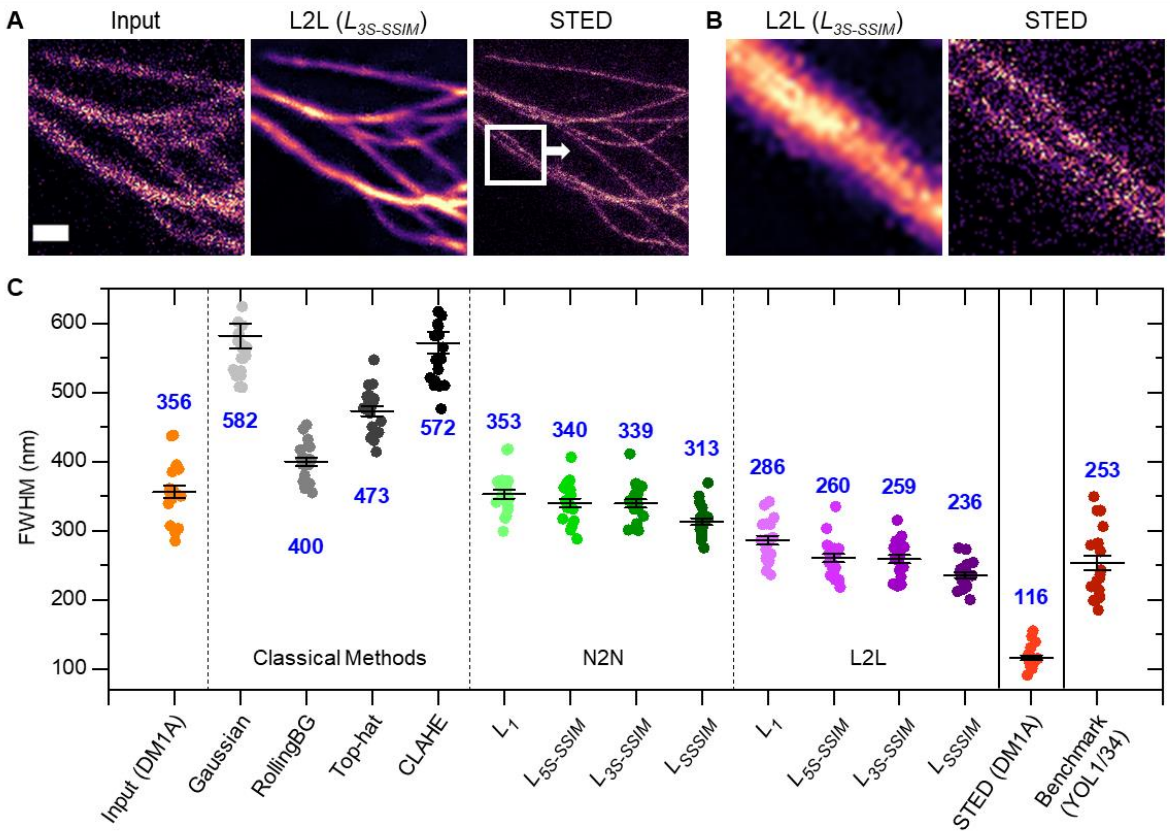

Fig. S4. Measured resolution in images of tubulin dependent on the image processing method.

(**A**) Confocal image of DM1A (tubulin), the prediction by a CNN after training with confocal images of DM1A/YOL1/34 as input/benchmark (see **Fig. S2**) using a *L3S-SSIM* loss function, and the corresponding STED image (scale bar = 1 µm). (**B**) Region of interest of the L2L result and STED image in (**A**). Close structures that are not resolved with confocal microscopy collapse into one microtubule in the prediction of a network.

(**C**) Extracted full width at half maxima (FWHMs) for 20 line profiles across single microtubules in (*from-left-to-right*) confocal images of DM1A which are used as training input for L2L/N2N training, images of DM1A after applying classical image processing methods (see **Fig. S3**), restored images of DM1A by a CNN after N2N and L2L training with different loss functions, STED images of DM1A, and confocal images of YOL1/34 which were used as benchmark for L2L. Image resolution decreases after using (*grey*) classical image processing methods, while DL-based methods decrease the average extracted width of single microtubules increasingly, using a MS-SSIM loss function with decreasing scale (*M*). Here, (*pink*) L2L outperforms (*green*) N2N. The extracted FWHMs in predictions of DM1A after L2L training with a *LSSIM* are closest to FWHMs obtained with STED microscopy (see also (*blue*) the mean FWHMs in nm for each category).

Journal of Cell Science • Supplementary information

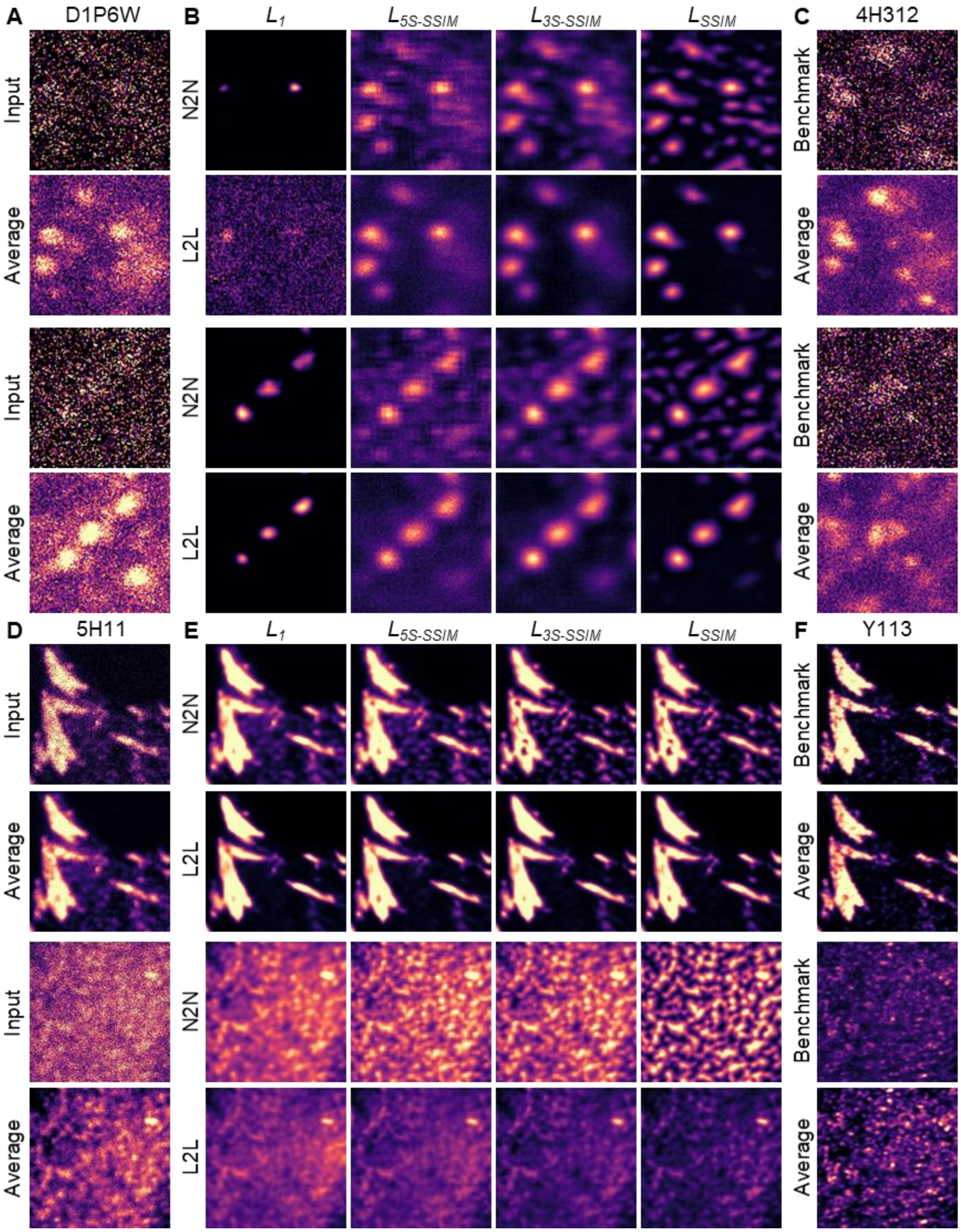

Fig. S5. Loss function-dependent label2label and noise2noise results for STED images of caveolae and confocal images of PXN.

(*Left*) Training inputs and corresponding 20-frame average images of (**A**) MeT5A cells labelled with D1P6W (CAVIN-1, an essential caveolae component) and (**D**) MeT5A cells labelled with 5H11 (PXN, an essential focal adhesion component). (**B**,**E**) Corresponding restorations by a CNN after L2L and N2N training with different loss functions. (*Right*) The benchmarks for L2L training and 20-frame average images for the (**C**) caveolae (4H312) or (**F**) PXN (Y113) dataset, respectively. The shown image pairs were excluded from the trainings.

Each image is contrast adjusted to (**A**,**C**) their 2nd and 100th percentile, or (**D**,**F**) contrast adjusted to the 2nd and 99.8th percentile of the respective input image (image dimensions: 1 µm x 1 µm (caveolae)/ 9.1 µm x 9.1 µm (PXN)).

Journal of Cell Science • Supplementary information

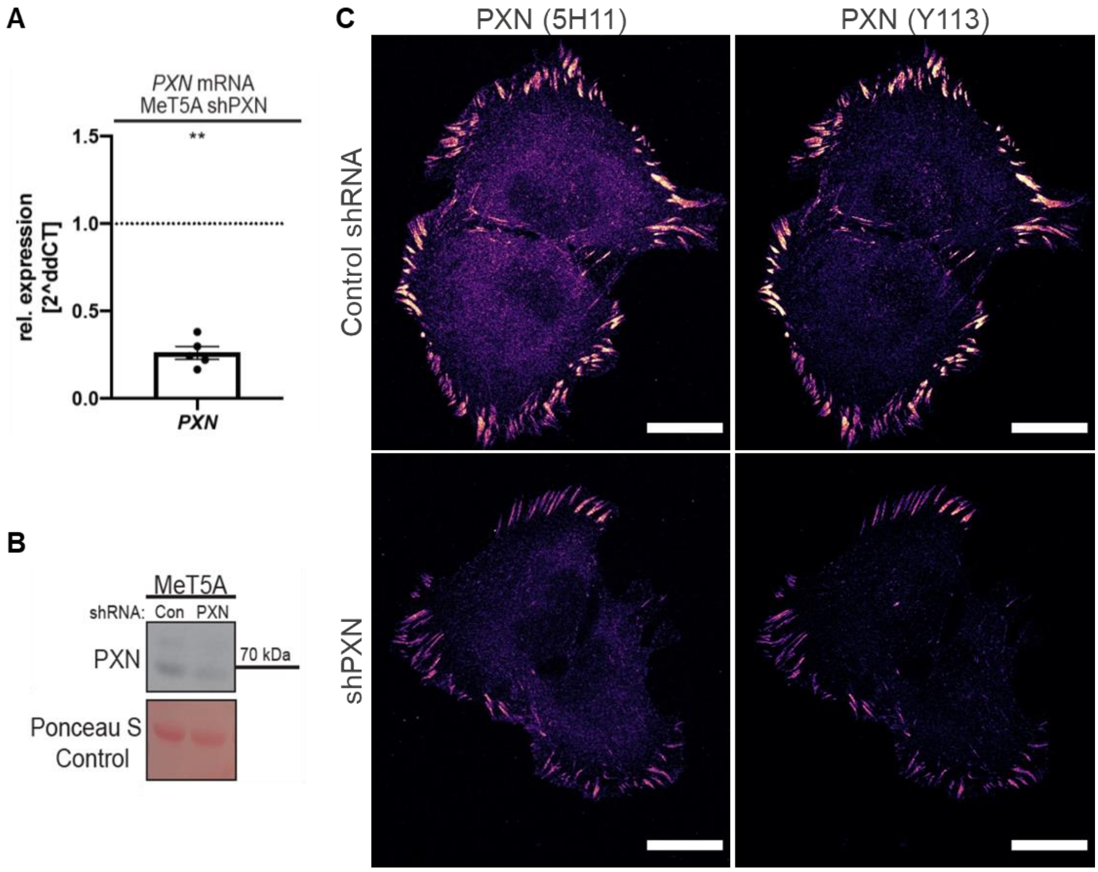

Fig. S6. *PAXILLIN* (*PXN*) knockdown in MeT5A cells.

PXN is a focal adhesion molecule. (**A**) Results of a RT-qPCR analysis, including the standard error of the mean: the relative expression of the *PXN* knockdown cells is 26.14±0.04%. (**B**) Western analysis of cell lysates of MeT5A cells visualising the PXN protein knockdown, with Ponceau stain ensuring equal loading. The monoclonal anti-PXN antibody Y113 was used. The PXN expression is reduced by around a half in the knockdown cells compared to the control. (**C**) Immunofluorescence images of MeT5A cells that were dual-labelled with the anti-PXN antibodies (*left*) 5H11 and (*right*) Y113 after (*top*) mock-infection and (*bottom*) shRNA mediated *PXN*-knockdown. The low signal of cytosolic protein in the knockdown cells for both antibodies indicate that the difference in cytosolic signal between the two, as observed in the control cells, originates from clone-dependent antibody binding to cytosolic PXN rather than unspecific binding (scale bar = 20 µm).

Journal of Cell Science • Supplementary information

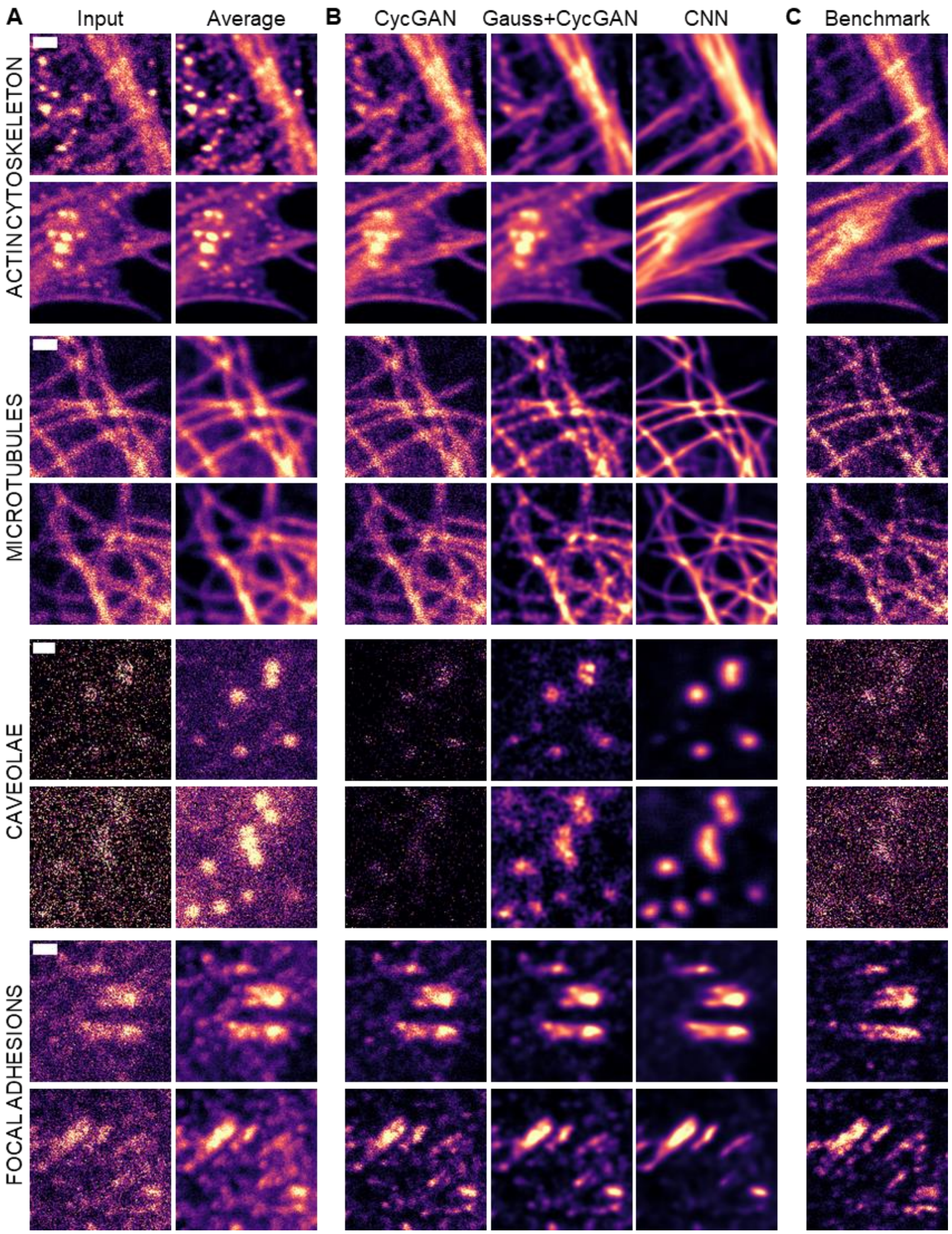

Fig. S7. Predictions by a CycleGAN and CNN after label2label training.

(**A**) Two representative inputs for L2L training and corresponding 20-frame average images of four different cellular structures (*top-to-bottom*): AC-15 (actin), DM1A (α-tubulin), D1P6W (CAVIN-1, an essential caveolae component), and 5H11 (PXN, an essential focal adhesion component). (**B**) Predicted images after training (*left-to-right*) a CycleGAN with unaligned image pairs without/with applying a Gaussian filter (σ=2) to the image patches prior to the network training, and predictions by a CNN after using a *L1* (PXN)/*L3S-SSIM* (all else) for the training. (**C**) Corresponding benchmarks for L2L (*top-to-bottom*): phalloidin (actin), YOL1/34 (α-tubulin), 4H312 (CAVEOLIN-1, an essential caveolae component), and Y113 (PXN). The CNN outperforms the CycleGAN in restoring images with enhanced contrast of the target structure (scale bar (*top-to-bottom*) = 1/1/0.2/1 µm).

Journal of Cell Science • Supplementary information

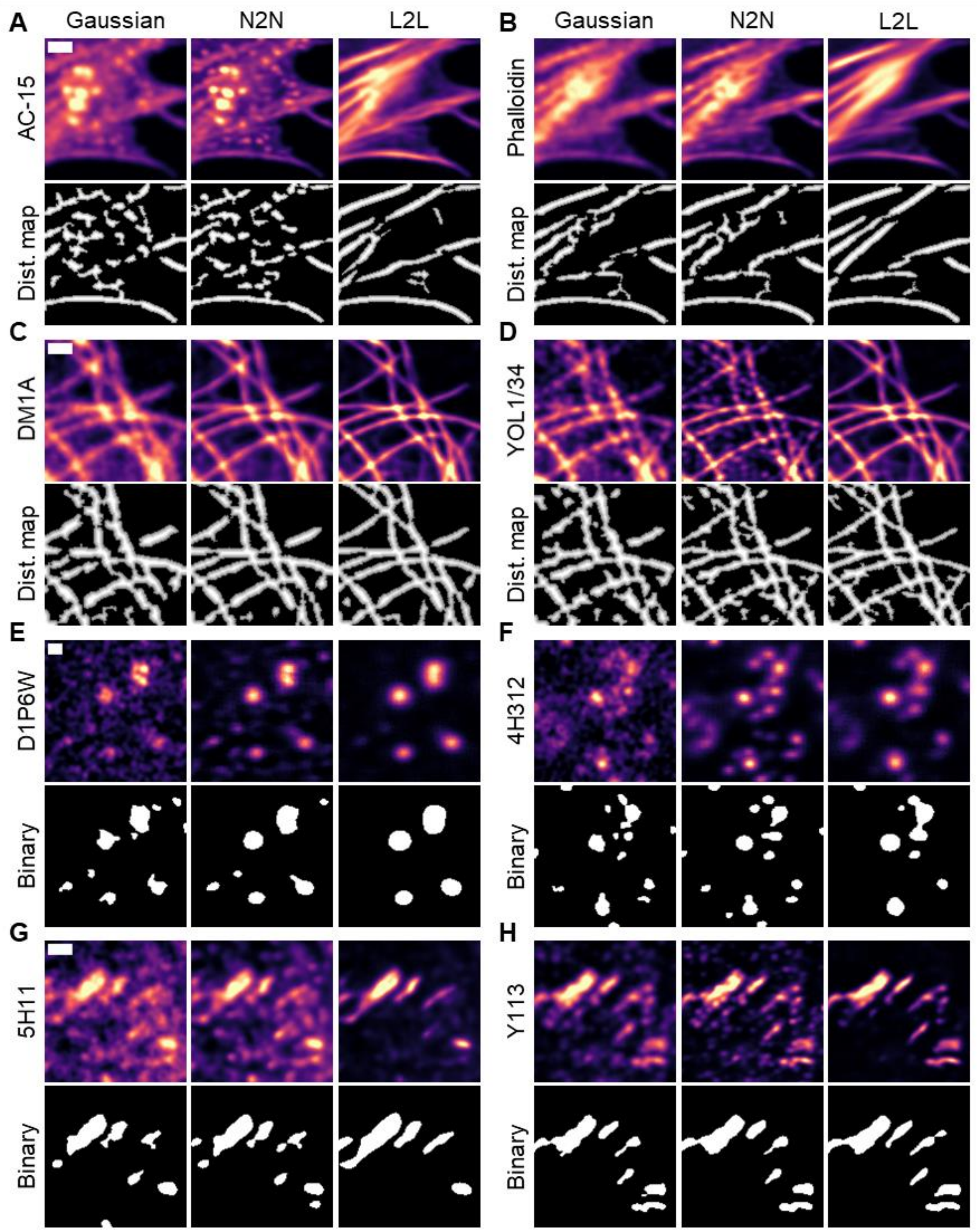

Fig. S8. Distance maps or binarisation of images dependent on pre-processing.
(*Top-to-bottom*) Images of dual-labelled (**A**,**B**) HeLa cells targeting the actin cytoskeleton, and MeT5A cells targeting the (**C**,**D**) microtubule network, (**E**,**F**) caveolae and (**G**,**H**) focal adhesions (PXN), respectively. For N2N, a CNN was trained with two noise realisations of the respective label. For L2L, images of (**A**,**B**) AC-15/Phalloidin, (**C**,**D**) DM1A/YOL1/34, (**E**,**F**) D1P6W/4H312, (**G**,**H**) 5H11/Y113 are used as input/benchmark for the training. For the network trainings, a L_1 (PXN)/$L_{3S\text{-}SSIM}$ (all else) loss function was used. (**A**-**D**) Distance maps or (**E**-**H**) binarised images are shown for the respective pre-processed images (see also **Fig. S7** for corresponding raw images). L2L facilitates post-processing due to reduced non-structural signal in the cell cytoplasm in the predictions (scale bar (*top-to-bottom*) = 1/1/0.2/1 µm).

Journal of Cell Science • Supplementary information

Table S1. Evaluation of classical methods to enhance image contrast versus the deep learning-based image restoration methods noise2noise and label2label.

Calculation of the average PSNR, NRMSE and MS-SSIM indices (*M* = 1, 3, 5) between (*top panel*) representative training input images, (*middle panel*) after applying a Gaussian filter, a Gaussian filter and rolling-ball background subtraction, a Gaussian filter and top-hat filter, or a Gaussian filter and contrast limited adaptive histogram equalization (CLAHE), or (*bottom panel*) predicted by a CNN after N2N/L2L training, and the images that are used as benchmark for L2L training with the (**A**) actin, (**B**) tubulin, (**C**) caveolae and (**D**) PXN (focal adhesion) dataset. For qualitative results see **Fig. S3**. On average, predicted images after L2L training exhibit, in comparison, the highest correlation to the respective benchmark images. The randomly selected image pairs (*N* = 1,000) to calculate the metrics were excluded from the network trainings.

**A**	*Actin Cytoskeleton*					**B** *Microtubule Network*				
	**PSNR (dB)**	**NRMSE**	**MS-SSIM**			**PSNR (dB)**	**NRMSE**	**MS-SSIM**		
			M=5	***M=3***	***M=1***			***M=5***	***M=3***	***M=1***
**Input**	16.73	0.149	0.437	0.333	0.150	17.23	0.140	0.547	0.365	0.139
**Gaussian**	18.25	0.127	0.526	0.439	0.254	18.31	**0.124**	0.627	0.435	0.180
**Rolling BG**	16.69	0.151	0.406	0.355	0.186	17.13	0.141	0.544	0.388	0.153
**Tophat**	16.55	0.153	0.403	0.342	0.173	17.00	0.144	0.535	0.370	0.144
**CLAHE**	18.01	0.130	0.525	0.435	0.247	18.05	0.127	0.631	0.441	0.182
**N2N**	17.99	0.131	0.528	0.444	0.258	**18.31**	0.124	0.647	0.459	0.196
**L2L**	**20.05**	**0.103**	**0.667**	**0.562**	**0.338**	18.27	0.124	**0.661**	**0.477**	**0.206**
**C**	*Caveolae*					**D** *PAXILLIN*				
**Input**	14.89	0.181	0.156	0.110	0.048	18.73	0.118	0.455	0.338	0.151
**Gaussian**	**15.05**	**0.178**	**0.172**	0.129	0.061	20.50	0.098	0.554	0.440	0.263
**Rolling BG**	14.91	0.181	0.162	0.119	0.052	20.41	0.100	0.548	0.443	0.280
**Tophat**	14.79	0.183	0.149	0.109	0.044	19.67	0.109	0.501	0.399	0.239
**CLAHE**	14.99	0.179	0.170	0.126	0.057	20.19	0.101	0.535	0.421	0.247
**N2N**	14.90	0.181	0.162	0.126	0.058	20.74	0.095	0.573	0.462	0.288
**L2L**	14.98	0.179	0.165	**0.134**	**0.066**	**21.31**	**0.091**	**0.600**	**0.494**	**0.328**
