# Supplementary material for: Label2label: training a neural network to selectively restore cellular structures in fluorescence microscopy
Source: J Cell Sci. 2022 Feb 10;135(3):jcs258994. doi: 10.1242/jcs.258994 (PMC8918818; doi:10.1242/jcs.258994)
Supplement: Reviewer comments [file joces-135-258994_review_history.pdf]

Label2label: Training a neural network to selectively restore cellular structures in fluorescence microscopy

Lisa Sophie Kölln, Omar Salem, Jessica Valli, Carsten Gram Hansen and Gail McConnell
DOI: 10.1242/jcs.258994

Editor: Jennifer Lippincott-Schwartz

Review timeline
Original submission: 24 June 2021
Editorial decision: 23 August 2021
First revision received: 28 October 2021
Accepted: 17 December 2021

Original submission

First decision letter

MS ID#: JOCES/2021/258994

MS TITLE: Label2label: Training a neural network to selectively restore cellular structures in fluorescence microscopy

AUTHORS: Lisa Sophie Kölln, Omar Salem, Jessica Valli, Carsten Gram Hansen, and Gail McConnell
ARTICLE TYPE: Research Article

We have now reached a decision on the above manuscript.

To see the reviewers' reports and a copy of this decision letter, please go to: https://submit-jcs.biologists.org and click on the 'Manuscripts with Decisions' queue in the Author Area. (Corresponding author only has access to reviews.)

As you will see, the reviewers gave favourable reports but raised some critical points that will require amendments to your manuscript. I hope that you will be able to carry these out because I would like to be able to accept your paper, depending on further comments from reviewers.

We are aware that you may be experiencing disruption to the normal running of your lab that makes experimental revisions challenging. If it would be helpful, we encourage you to contact us to discuss your revision in greater detail. Please send us a point-by-point response indicating where you are able to address concerns raised (either experimentally or by changes to the text) and where you will not be able to do so within the normal timeframe of a revision. We will then provide further guidance. Please also note that we are happy to extend revision timeframes as necessary.

Please ensure that you clearly highlight all changes made in the revised manuscript. Please avoid using 'Tracked changes' in Word files as these are lost in PDF conversion.

I should be grateful if you would also provide a point-by-point response detailing how you have dealt with the points raised by the reviewers in the 'Response to Reviewers' box. Please attend to all of the reviewers' comments. If you do not agree with any of their criticisms or suggestions please explain clearly why this is so.

© 2022. Published by The Company of Biologists under the terms of the Creative Commons Attribution License (https://creativecommons.org/licenses/by/4.0/).

Reviewer 1

Advance summary and potential significance to field

The authors describe Label2Label: a deep learning-based image restoration algorithm for microscopy that is trained using redundancies between images acquired with biologically distinct labels for increasing structural contrast.
This method is conceptually very interesting, as it represents a shift from denoising methods based on classical properties of images (e.g. signal-to-noise ratio, local noise statistics) to denoising based on the inherent imperfections of biological labelling. There is also technical novelty in the exploration of an MS-SSIM based loss function used in training the network.

There are many strengths to this work. The authors elegantly state the limitation of many deep learning-based image restoration methods, in that they disregard intrinsic properties of biological fluorescence microscopy (lines 70-79); this is in general under-discussed in the field. The actin data (Figure 1) is very compelling in favour of L2L as an improvement on current state-of-the art de-noising. The knockdown validation experiment for the paxillin data (SI Figure 4) was also a very interesting component of the paper, as it underscores where ‘unwanted’ signal arises from in biological images, and provokes thought on what signal we actually want to keep from our original images. The demonstration of the network is separating signals from two superposed structures (Figure 4) could be very powerful for multicolour imaging with spectrally overlapping fluorophores and I think this would be generally very useful in the fluorescence microscopy community. The authors also have a firm grasp on the shortcomings of image quality assessment metrics for fluorescence microscopy (lines 435-445).

One of the most interesting things about the paper was that while reading it, I was immediately thinking of other contexts that L2L could be used in (could a CycleGAN-based method be used to restore between the same structure disrupted under different fixation conditions? could L2L be trained on a dataset with GFP and anti-GFP immunolabelling for restoration of live-cell data?), which is a real credit to the authors and the appeal of the work. (note that my questions do not need answering here, just examples to demonstrate that I found the work exciting!)

Comments for the author

The authors describe Label2Label: a deep learning-based image restoration algorithm for microscopy that is trained using redundancies between images acquired with biologically distinct labels for increasing structural contrast. This method is conceptually very interesting, as it represents a shift from denoising methods based on classical properties of images (e.g. signal-to-noise ratio, local noise statistics) to denoising based on the inherent imperfections of biological labelling. There is also technical novelty in the exploration of an MS-SSIM based loss function used in training the network.

There are many strengths to this work. The authors elegantly state the limitation of many deep learning-based image restoration methods, in that they disregard intrinsic properties of biological fluorescence microscopy (lines 70-79); this is in general under-discussed in the field. The actin data (Figure 1) is very compelling in favour of L2L as an improvement on current state-of-the art de-noising. The knockdown validation experiment for the paxillin data (SI Figure 4) was also a very interesting component of the paper, as it underscores where 'unwanted' signal arises from in biological images, and provokes thought on what signal we actually want to keep from our original images. The demonstration of the network is separating signals from two superposed structures (Figure 4) could be very powerful for multicolour imaging with spectrally overlapping fluorophores, and I think this would be generally very useful in the fluorescence microscopy community. The authors also have a firm grasp on the shortcomings of image quality assessment metrics for fluorescence microscopy (lines 435-445).

One of the most interesting things about the paper was that while reading it, I was immediately thinking of other contexts that L2L could be used in (could a CycleGAN-based method be used to restore between the same structure disrupted under different fixation conditions? could L2L be trained on a dataset with GFP and anti-GFP immunolabelling for restoration of live-cell data?),

© 2022. Published by The Company of Biologists under the terms of the Creative Commons Attribution License (https://creativecommons.org/licenses/by/4.0/).

which is a real credit to the authors and the appeal of the work. (note that my questions do not need answering here, just examples to demonstrate that I found the work exciting!)

I have a few general concerns that I would like the authors to address:

Application of MS-SSIM metric
There are quite a few parameters involved in the MS-SSIM calculation. For example, how did the authors select the values of the weights ? In the Methods, the authors use the weights for M=5 as described in reference 32, but these were originally derived (as far as I can tell) from human perceptual assessment of 64x64 pixels distorted images of subjects such as faces, natural scenes etc., which is a very different dataset to the images here. I am definitely not asking the authors to try and derive a microscopy-specific set of weights, but I am curious to know how sensitive the MS-SSIM loss function and assessment of images using MS-SSIM is to these weights. For example, are radically different results obtained if L2L is trained with a MS-SSIM loss function where the weights are all equivalent, or where higher frequency components have larger weights etc? On a similar note, how was the size of the low-pass Gaussian filter selected? This feels like a parameter which should depend on the pixel sampling size and image resolution and thus vary between images.
Is there any way of using a priori information about the structure being imaged (such as the typical structure size, or dimensionality) to decide which value of M should be used in the L2L loss function? It was interesting to observe that M=5 yielded the best results for actin, tubulin, caveolae, and paxillin) which all exist on similar size scales, yet M=1 yielded the best result for the Sytox nuclear stain, which is a much larger structure that is also two- dimensional in the image.

Comparison with 'classical' denoising methods
Deep learning is clearly a very powerful technique for image processing, but it would be interesting to see how L2L and N2N compare to simpler techniques such as Gaussian smoothing (which could recreate the 'in-painting' discussed by the authors) and a rolling ball background subtraction (which may recreate the cytosolic signal filter for the paxillin data). This is already shown to a small extent in the Gaussian-filtered images in SI Figure 5, but I would like to be thoroughly convinced that the image content in L2L-processed data is more useful than just applying a very simple filter that has no danger of inducing hallucination artefacts. I would like to see quality metrics for such non-deep learning methods shown alongside the L2L and N2N results.
On a related point, what is the interplay between contrast increase/denoising and image resolution (as this is a disadvantage of just using a Gaussian filter, for example)? And does the order of the MS-SSIM make a difference to resolution? I mainly ask this because in the microtubule data that is validated with the STED imaging (Figure 2A, lower row), the microtubule diameters appear thinner in the L2L 3S-SSIM image than the L1 image.

Image artefacts
I have a concern with the data in the lower row of Figure 2A that all three deep learning methods appear to be collapsing structures onto one another (artificial sharpening) - this is most visible on the triangle-shaped crossing halfway up the image on the right-hand side, and on the two parallel microtubules running diagonally across the top left. It is really useful to have the STED validation here, as otherwise this sort of artefact would not be obvious. In terms of biological information, although the input image clearly looks awful, I wonder whether this is still actually as reliable than the denoised data (e.g. if I had to manually trace the microtubules in both the input and L2L 3S-SSIM images, I think I would get results that I was equally confident in from both).
When the authors mention hallucinations in images, it would be good to annotate examples of these either directly on the images or in a supplementary figure (to avoid obscuring the main figures too much). Examples are mentioned in lines 209-212 and lines 283-285, and it would be useful to guide the reader a little more here, especially if they are unfamiliar with looking at deep learning-generated images.

Implementation/deployment of algorithm
Is the code freely available for people to download and use on their own data? Would any of the trained networks here be directly applicable to someone else's data, or should new networks be

© 2022. Published by The Company of Biologists under the terms of the Creative Commons Attribution License (https://creativecommons.org/licenses/by/4.0/).

trained for each use instance? If the code is not available, or would not be practical to be used directly on fresh data from other sources, I personally think it is acceptable to just say that the work here is a useful proof of principle (as the manuscript is currently presented I am not sure if this is something that I can instantly use myself or not). If this method is already intended to be used by researchers, then there should also be guidance on which loss function is best to use - should this be a purely metric-based decision as shown in Table 1, or more subjective and based on the user's judgement (e.g. as in lines 335-337, also discussed in lines 435-436)?

A few smaller miscellaneous comments:

- The description of CycleGANs (lines 60-62) is a little confusing, especially for a biological audience (e.g. what is back-translation?)
- In the description of noise2clean (lines 95-99) it might be worth also highlighting reference 8 by the name 'CARE', as I think this is the name by which most people know it as. Also, I did not quite follow the argument against using noise2clean in IF data with non-dynamic specimens and high photon counts.
- In Table 1, evaluation metrics such as NRMSE can sometimes be a bit misleading because of good matching between large regions of background which dominate an image, rather than the structure itself. SSIM lends itself to creating a map of similarity via its sliding window implementation - would it be possible for one of the datasets to show such a map (in addition to the RMS map as in Figure 1) given the emphasis on using MS-SSIM?
- Is there a difference if you switch the input and benchmark images in training, or is the method somewhat commutative? If there is a difference, and the two labels are not of markedly different quality (as appears the case at least for the microtubules data), could this switching be used as an additional data augmentation? In my experience, many different markers/antibodies for the same structure have been similarly mediocre (i.e. it wouldn't be clear which to use as the benchmark)
- I did not understand lines 424-426, sorry (what is a non-dynamic image corruption?)
- To show that L2L is useful, it would be good to actually demonstrate the binarization discussed in lines 466-467 in a Supplementary Figure, comparing L2L with just e.g. a big Gaussian blur or threshold. This would be the cherry on the cake to demonstrate how L2L can be used to help downstream analysis rather than just make more visually appealing images.

<u>Reviewer 2</u>

Advance summary and potential significance to field

The key advance or contribution of this paper is that the authors demonstrate a new application of standard CNN models which can help assist or accelerate quantitative cell biology findings.

Comments for the author

In general, I think the paper is well written and indeed demonstrates a new application of a CNN in helping accerlerate quantitative cell biology study. But I do have two major concerns, which in my opinion must be addressed before publication.

Major concern 1:
The paper's claim on what the model is actually doing is not accurate, and need some re-wording. The paper claims that "..., but current image restoration methods cannot correct for background signals originating from the label. Here, we report a new method to train a CNN as content filter for non-specific signals in fluorescence images that does not require a clean benchmark, using dual-labelling to generate the training data". But, there is no strong evident showing the proposed L2L model actually correct the backgroung signals originating from labels and only filter out the target content. What the L2L model actually does is simply a "style transfer", namely transferring the image from one type of signals or styles to another, via the training using dual-labelling images. For example, the paper mentions that "While the phalloidin stain labels almost exclusively the actin filaments, images of the antibody (AC-15) exhibit a high background signal in the cell body. This background signal likely originates from unspecific

© 2022. Published by The Company of Biologists under the terms of the Creative Commons Attribution License (https://creativecommons.org/licenses/by/4.0/).

binding and/or binding to cytosolic protein by the AB, resulting in high intensity punctate regions as observed in the cell cytoplasm". We can see that the model is simply transfering one type of signal (AC-15) with high background signal to another type of signals (phalloidin) with much less background signals. The background singals or noise still exist, but just transformed from one type of background signal to another. I would suggest just to avoid explicitly claiming filtering out the struture signal or correcting background signals, instead saying something like transferring one label to another to make the structure more visible or something similar.

Major concern 2:
Are all structures able to be imaged via dual-labelling images? If not, I think this needs to clearly noted in the paper and more importantly claimed as one disadvantage over other methods like N2N which has no such requirement.

Minor concerns:
- why cycleGAN is only tested on FA structures?
- I would highly recommend to show some not quite successful ROI in the predictions. For example, in Figure 1, I can clearly see some not quite good ROI in the predictions, but the zoom-in areas are more like good ROI examples.
- I would highly recommend to do another type of validation. Specially, I would recommend to do segmentation on the cleaner images and the predictions, as well as the noiser images to show that after restoration the images can be better segmented to permit more accruate downstream quantitative analysis. For example for the actin example, segmentation from AC-15 can be hard, but segmentation from Phalloidion might be much easier so that the actual topology of the actin structures can be better identified. It would be good to show the segmentation from prediction is comparable to segmenataino from Phalloidion and better permits accurate downstream analysis comparig to AC-15.

First revision

Author response to reviewers' comments

We thank both reviewers for their thorough and helpful feedback and comments.

Reviewers' comments in **Blue**, our response in **Black**.

Our response to comments from Dr Culley:

The authors describe Label2Label: a deep learning-based image restoration algorithm for microscopy that is trained using redundancies between images acquired with biologically distinct labels for increasing structural contrast. This method is conceptually very interesting, as it represents a shift from denoising methods based on classical properties of images (e.g. signal-to-noise ratio, local noise statistics) to denoising based on the inherent imperfections of biological labelling. There is also technical novelty in the exploration of an MS-SSIM based loss function used in training the network.

There are many strengths to this work. The authors elegantly state the limitation of many deep learning-based image restoration methods, in that they disregard intrinsic properties of biological fluorescence microscopy (lines 70-79); this is in general under-discussed in the field. The actin data (Figure 1) is very compelling in favour of L2L as an improvement on current state-of-the art de-noising. The knockdown validation experiment for the paxillin data (SI Figure 4) was also a very interesting component of the paper, as it underscores where ‘unwanted’ signal arises from in biological images, and provokes thought on what signal we actually want to keep from our original images. The demonstration of the network is separating signals from two superposed structures (Figure 4) could be very powerful for multicolour imaging with spectrally overlapping fluorophores, and I think this would be generally very useful in the fluorescence microscopy community. The authors also have a firm grasp on the shortcomings of image quality assessment metrics for fluorescence microscopy (lines 435-445).

© 2022. Published by The Company of Biologists under the terms of the Creative Commons Attribution License (https://creativecommons.org/licenses/by/4.0/).

One of the most interesting things about the paper was that while reading it, I was immediately thinking of other contexts that L2L could be used in (could a CycleGAN-based method be used to restore between the same structure disrupted under different fixation conditions? could L2L be trained on a dataset with GFP and anti-GFP immunolabelling for restoration of live-cell data?), which is a real credit to the authors and the appeal of the work. (note that my questions do not need answering here, just examples to demonstrate that I found the work exciting!)

We thank Dr Culley for the kind comments about our work. We do think that images for the L2L training do not have to stem from the same sample if a CycleGAN is used for the training. This might indeed be useful, for example, in multiplex imaging experiments were, as pointed out, certain fixatives might lead to increased unspecific cytosolic background signals for some labels, but are essential to study other targets in a multi-labelled sample (as known, for example, in fixatives that contain glutaraldehyde). Applying L2L to retrospectively enhance live cell data that traditionally suffers from high background signals and high image noise is another interesting prospect.

I have a few general concerns that I would like the authors to address:

Concern 1: Application of MS-SSIM metric: There are quite a few parameters involved in the MS-SSIM calculation. For example, how did the authors select the values of the weights γ_i? In the Methods, the authors use the weights for M=5 as described in reference 32, but these were originally derived (as far as I can tell) from human perceptual assessment of 64x64 pixels distorted images of subjects such as faces, natural scenes etc., which is a very different dataset to the images here. I am definitely not asking the authors to try and derive a microscopy-specific set of weights, but I am curious to know how sensitive the MS-SSIM loss function and assessment of images using MS-SSIM is to these weights. For example, are radically different results obtained if L2L is trained with a MS-SSIM loss function where the weights are all equivalent, or where higher frequency components have larger weights etc? On a similar note, how was the size of the low-pass Gaussian filter selected? This feels like a parameter which should depend on the pixel sampling size and image resolution and thus vary between images.

Thank you for your question. Determining weights for a MS-SSIM index specifically for fluorescence microscopy images is challenging, since immunofluorescence (IF) images traditionally exhibit many low intensity pixels. This leads to a much narrower image histogram compared to the example images used by Wang et *al*. (2003) and makes a human perceptual assessment of image distortions as demonstrated by them challenging. Further, for our purpose (L2L training), it is unclear if weights should be determined empirically for the raw, noisy images of either label or, for example, for high frame average images instead (which in the case of, for instance, the caveolae dataset are still noisy).

Notably, the weight over scale trend for the "original" 5S-SSIM index follows the contrast sensitivity function of the human visual system. Therefore, to estimate the weights for a 3S-SSIM index, we fitted a polynomial function to the weights of the 5S-SSIM index over the scale using Origin (see line plot and fit results in **Figure R1**). We estimated the weights for the 3S-SSIM index by calculating the weights for x=1.25, 2.75, 4.25, using the fit function. Then those weights were normalised to sum up to 1, resulting in (0.2096, 0.4659, 0.3245).

© 2022. Published by The Company of Biologists under the terms of the Creative Commons Attribution License (https://creativecommons.org/licenses/by/4.0/).

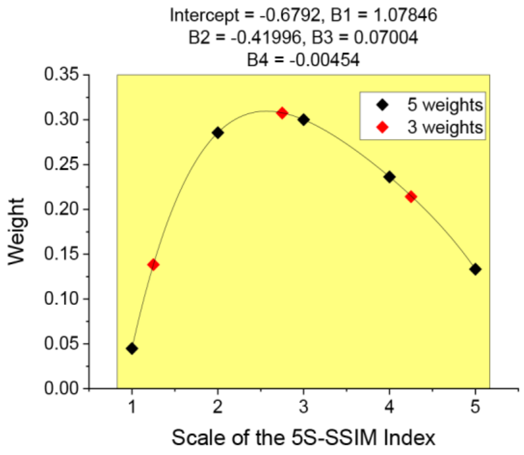

Figure R1 - Weight over scale for a MS-SSIM index
*(**Black**) Empirically determined weights for the 5S- SSIM index by Wang et al. (2003), (line plot) polynomial fit, and (**red**) weights used to calculated the 3S-SSIM index in this work prior to normalisation.*

We are satisfied with the predictions after using both, the 5S-SSIM and the 3S-SSIM loss function, with weights that follow the trend of the contrast sensitivity function and the filter sizes 7 or 11, respectively. Comparing the predictions dependent on the loss function, we observe the expected (see **Figure S2+S5**): with higher *M*, predictions converge towards results obtained when using a *L1* loss function for the network training instead.

Nonetheless, it is indeed a good question how sensitive the MS-SSIM index is to the weights and the filter size - and if that sensitivity is dependent on the particular dataset. We show this in our analysis below in **Table R1+R2** and **Figure R2.** In **Table R1+R2**, the calculated 3S- and 5S-SSIM indices are shown between 1000 randomly selected training inputs and benchmarks for L2L training for each dataset, using weights that follow different trends. While weights vary significantly (e.g. weights linearly increase or decrease), all calculated MS-SSIM indices deviate, on average, by less than ± 10% from the (arbitrary) mean. Further, the default weights used in this work lead to comparably high calculated 3S-/5S-SSIM indices for all datasets, with the caveolae dataset being the only exception.

	Weight of scale			3S-SSIM					
Trend	1	2	3	Actin	Tubulin	Caveolae	PXN	SYTOX	CD44
linear ↑	0.17	0.33	0.5	0.314	**0.385**	0.084	0.321	0.429	0.825
linear ↓	0.5	0.33	0.17	0.314	0.285	**0.160**	0.321	**0.585**	0.804
const.	0.33	0.33	0.33	0.311	0.331	0.114	0.319	0.499	0.814
triangle ↑	0.25	0.5	0.25	0.339	0.351	0.126	0.342	0.538	0.826
triangle ↓	0.4	0.2	0.4	0.292	0.316	0.105	0.302	0.470	0.804
"gaussian" ↑	0.17	0.67	0.17	**0.37**	0.373	0.140	**0.368**	0.581	**0.838**
"gaussian" ↓	0.44	0.11	0.44	0.279	0.307	0.100	0.292	0.452	0.798
default	0.21	0.47	0.32	0.333	0.365	0.110	0.338	0.502	0.827
			MEAN	0.319	0.339	0.117	0.325	0.507	0.817
			STD	0.027	0.033	0.022	0.022	0.054	0.013

Table R2 - Weight-dependent 3S-SSIM indices for all generated datasets.
The indices were calculated between the images that were used as input and benchmark for L2L training for the particular dataset, or between the separate and superposed IF images of cells that were dual-labelled with a SYTOX stain and anti-CD44 antibody, respectively. For the calculation, 1,000 image patches were randomly selected from the training data. The size of the Gaussian filter was set to 11 (=default). The highest calculated 3S-SSIM indices for each dataset are depicted in bold.

© 2022. Published by The Company of Biologists under the terms of the Creative Commons Attribution License (https://creativecommons.org/licenses/by/4.0/).

Trend	Weight of scale 1	2	3	4	5	5S-SSIM Actin	Tubulin	Caveolae	PXN	SYTOX	CD44
linear ↑	0.07	0.13	0.2	0.27	0.33	0.406	**0.581**	0.141	0.446	0.442	0.801
linear ↓	0.33	0.27	0.2	0.13	0.07	0.374	0.378	**0.187**	0.391	**0.614**	0.790
const.	0.2	0.2	0.2	0.2	0.2	0.386	0.468	0.159	0.416	0.517	0.794
triangle ↑	0.11	0.22	0.33	0.22	0.11	0.427	0.515	0.162	0.444	0.565	0.813
triangle ↓	0.08	0.25	0.33	0.25	0.08	**0.439**	0.528	0.164	0.453	0.582	**0.819**
"gaussian" ↑	0.31	0.15	0.08	0.15	0.31	0.345	0.419	0.158	0.386	0.465	0.773
"gaussian" ↓	0.27	0.18	0.09	0.18	0.27	0.357	0.433	0.158	0.395	0.481	0.780
default	0.04	0.29	0.3	0.24	0.13	0.437	0.547	0.156	**0.455**	0.551	0.818
					MEAN	0.396	0.484	0.161	0.423	0.527	0.798
					STD	0.034	0.066	0.012	0.028	0.057	0.016

Table R2 – Weight-dependent 5S-SSIM indices for all generated datasets
The indices were calculated between the images that were used as input and benchmark for L2L training for the particular dataset, or between the separate and superposed IF images of cells that were dual-labelled with a SYTOX stain and anti-CD44 antibody, respectively. For the calculation, 1,000 image patches were randomly selected from the training data. The size of the Gaussian filter was set to 7. The highest calculated 5S-SSIM indices for each dataset are depicted in bold.

Notably, for the caveolae dataset, the highest and lowest 3S- and 5S-SSIM indices are observed for weights that linearly decrease and increase, respectively. For the tubulin dataset, this trend is the opposite.

Regarding the filter size: The default filter size when calculating the SSIM/MS-SSIM is 11 px (Wang et *al.* 2003). This was also the filter size we used for the SSIM and 3S-SSIM loss function. However, due to the (M-1) times downsampling of the image patches during the calculation of the MS-SSIM index, the maximal possible filter size is the size of the images in the last iteration, which (for image pairs of size 128 px x 128 px) is 8 px x 8 px in size. Consequently, we reduced the filter size in a 5S-SSIM loss function to 7.

In **Figure R2A**, the calculated 3S- and 5S-SSIM indices are shown between randomly selected training inputs and benchmarks for L2L training for each dataset, using different sizes for the Gaussian filter and the default weights. In **Figure R2B**, the results are shown for (*left*) the caveolae and (*right*) the tubulin dataset, using default, linearly decreasing or linearly increasing weights for the calculations (see also **Table R1+R2**).

© 2022. Published by The Company of Biologists under the terms of the Creative Commons Attribution License (https://creativecommons.org/licenses/by/4.0/).

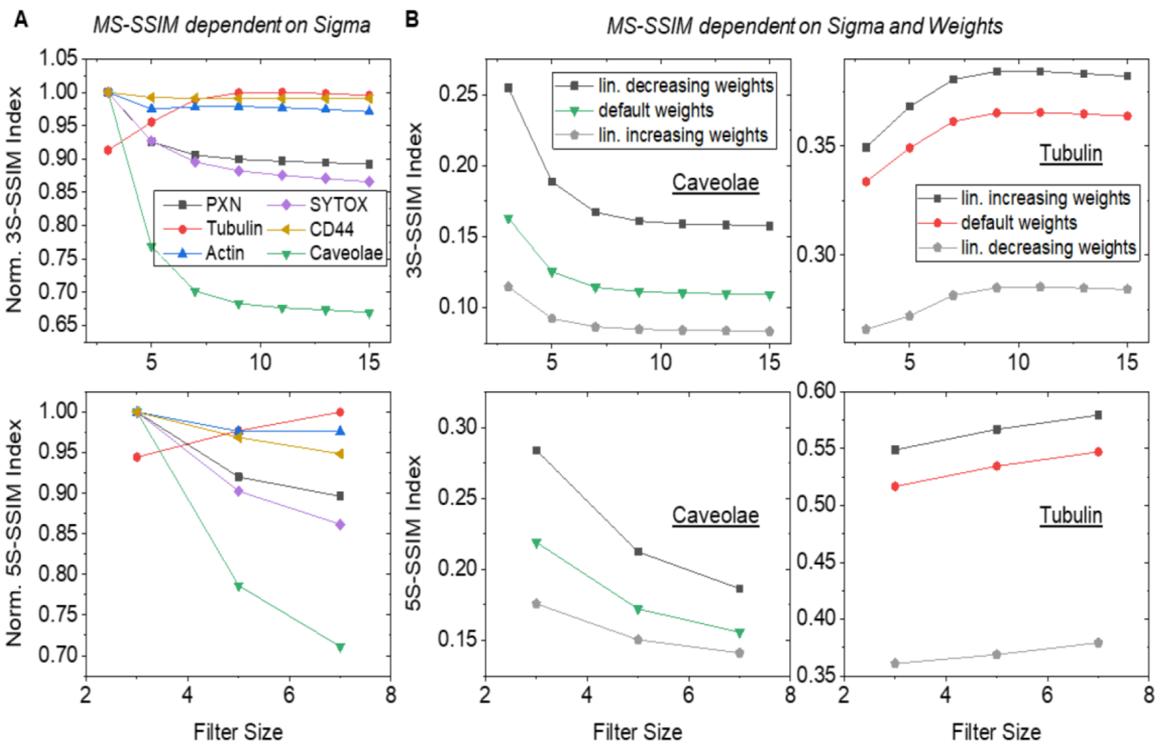

Figure R2 - Calculated MS-SSIM indices for different sizes of the Gaussian filter
The (**top**) *3S-SSIM and* (**bottom**) *5S-SSIM indices were calculated between the images that were used as input and benchmark for L2L training for the particular dataset, or between the separate and superposed IF images of cells that were dual-labelled with a SYTOX stain and anti-CD44 antibody, respectively. For that, 1,000 image patches were randomly selected from the training data. For the calculation,* (**A**,**B**) *default weights,* (**B**) *linearly decreasing, and* (**B**) *linearly increasing weights were used (see also* **Table R1+R2***).*

We make the following observations: (1) For most datasets, the MS-SSIM index changes only slightly with filter size; here, indices calculated for the caveolae dataset show the highest filter size- dependency. (2) Only for the tubulin dataset, we observe an increase in index for higher filter sizes, while for all other datasets, this trend is reversed. (3) The trend for each dataset seems to be independent of the selected weights (see **Figure R2**, *right*).

To answer the question, how much those parameters influence the predictions of a CNN after L2L training, we trained the CNN with a 3S-SSIM loss function, using different weights and filter sizes. We chose the tubulin and caveolae dataset as examples, since both showed the same weight-dependent trend in the respective 3S- and 5S-SSIM indices (compare **Table R1+R2**), and deviated most from the observed filter size-dependent trend across all datasets (see **Figure R2**). In **Figure R3**, we show the qualitative results of those trainings.

For the tubulin dataset, we find that differences between the predictions are very minor (see **Figure R3**). For the caveolae dataset, a weak trend is observable: at smaller filter sizes, structures are restored slightly less blurry. However, caveolae that appear with low intensity in the input image are restored more successfully when using a higher filter size.

© 2022. Published by The Company of Biologists under the terms of the Creative Commons Attribution License (https://creativecommons.org/licenses/by/4.0/).

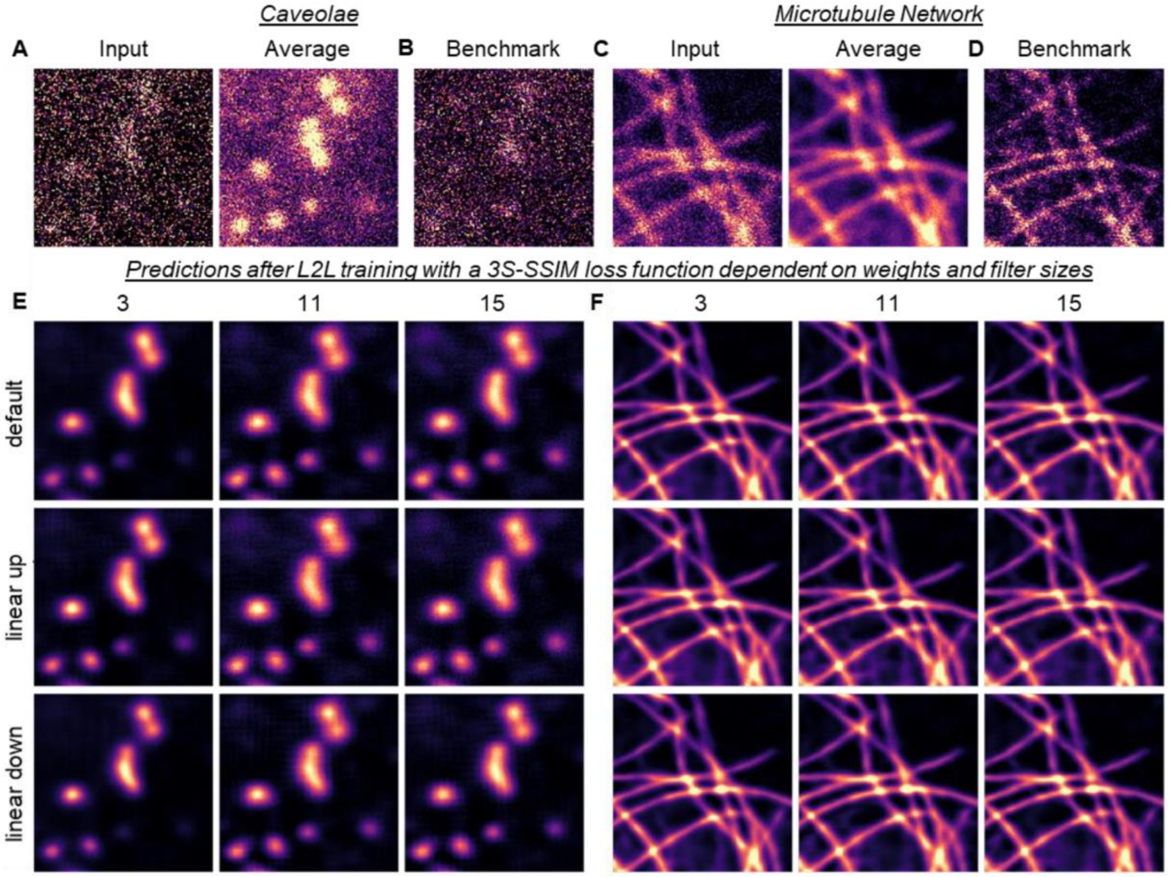

Figure R3 – Predictions of a CNN after L2L training, using a 3S-SSIM loss function with different sizes of the Gaussian filter and weights

(**A**) *Input (D1P6W, recognising the essential caveolae component CAVIN-1), corresponding 20-frame average, and* (**B**) *benchmark image (4H312, recognising the essential caveolae component CAVEOLIN-1) for L2L training for the caveolae dataset.* (**C**) *Input (DM1A), corresponding 20-frame average, and* (**D**) *benchmark image (YOL1/34) for L2L training with the tubulin dataset.* (**E**,**F**) *Restorations of a CNN after L2L training with a 3S-SSIM loss function, using a size of* (left-to-right) *3, 11 (=default) or 15 for the Gaussian filter, and weights that* (top-to-bottom) *follow the contrast sensitivity function (=default), linearly increase or linearly decrease with the iteration (image dimensions: 1.3 µm x 1.3 µm (caveolae)/ 5.8 µm x 5.8 µm (tubulin)).*

Since, overall, qualitative L2L results vary only slightly for different weights and filter sizes, and space is limited in the manuscript, we exclude these findings in the manuscript. However, we added the following explanation:

New<br><br>151-153	Weights of the MS-SSIM loss function were selected such that they follow the contrast sensitivity function of the human visual system (Wang, Simoncelli and Bovik, 2003).

old<br><br>614-616	For a *L3S-SSIM*, the weights were set to (0.2096, 0.4659, 0.3245), for a *L5S-SSIM*, the filter size for the Gaussian filter was set to 7; otherwise the suggested settings in [32] were used.

© 2022. Published by The Company of Biologists under the terms of the Creative Commons Attribution License (https://creativecommons.org/licenses/by/4.0/).

New<br><br>612-615	For a *L3S-SSIM*, the weights were set to (0.2096, 0.4659, 0.3245), for a *L5S-SSIM*, the size for the Gaussian filter was set to 7, which is the maximum possible filter size for the selected patch size; otherwise the suggested settings in (Wang, Simoncelli and Bovik, 2003) were used.

Is there any way of using a priori information about the structure being imaged (such as the typical structure size, or dimensionality) to decide which value of M should be used in the L2L loss function? It was interesting to observe that M=5 yielded the best results for actin, tubulin, caveolae, and paxillin) which all exist on similar size scales, yet M=1 yielded the best result for the Sytox nuclear stain, which is a much larger structure that is also two dimensional in the image.

Unfortunately, in our experience, selecting the right scale for a MS-SSIM loss function is only possible through trial. However, the results for our datasets that all target quite different cellular structures suggest: (a) a high-scale SSIM loss function is always advantageous to a single SSIM loss function; and (b) in cases where the to-filter-out signal deviates too little between the input and the benchmark, the more conservative L1 loss function might be a better choice to avoid the artificial accentuation of non- structural signal in the predictions (please see 2nd paragraph in discussion).

Concern 2: Comparison with 'classical' denoising methods: Deep learning is clearly a very powerful technique for image processing, but it would be interesting to see how L2L and N2N compare to simpler techniques such as Gaussian smoothing (which could recreate the 'in-painting' discussed by the authors) and a rolling ball background subtraction (which may recreate the cytosolic signal filter for the paxillin data). This is already shown to a small extent in the Gaussian-filtered images in SI Figure 5, but I would like to be thoroughly convinced that the image content in L2L-processed data is more useful than just applying a very simple filter that has no danger of inducing hallucination artefacts. I would like to see quality metrics for such non-deep learning methods shown alongside the L2L and N2N results.

Thank you for raising this issue. We now include a comparison of L2L results to a number of classical image processing methods in **new Figure S3 and Table S1 in the SI**.

We have also changed the manuscript as follows:

old<br><br>252-253	Also, both methods outperform the corresponding 20-frame average image (see **Figure 2A** (*top*) and **SI Figure 2**).
new 241-<br><br>243	Here, both methods outperform the corresponding 20-frame average image (see **Fig. 2A** (*top*) and **Fig. S2D**,**F**), and classical image processing methods like a Gaussian or top- hat filter (see **Fig. S3** and **Table S1**).

old<br><br>467-468	Here, the systematic recovery of specific structure by the network can make L2L superior to classical image processing methods.
New<br><br>458-460	Notably, the systematic recovery of specific structure and the adaptability of L2L to images of a multitude of targets potentially makes L2L superior to classical image processing methods (as shown in **Fig. S3** and **Table S1**).

© 2022. Published by The Company of Biologists under the terms of the Creative Commons Attribution License (https://creativecommons.org/licenses/by/4.0/).

New<br><br>646-654	Image processing and analysis was conducted in Python utilising the following functions/libraries in default if not stated otherwise. To compare L2L with classical image processing methods (see **Fig. S3** and **Table S1**), the following steps were undertaken for images of actin/tubulin/caveolae/PXN: Gaussian filters were applied with a sigma of 2/2/3/2 using ndimage.gaussian_filterin scipy (Virtanen *et al.*, 2020);
methods	for rolling-ball background (BG) subtraction, subtract_background_rolling_ball from https://github.com/mbalatsko/opencv-rolling-ball was used with a radius of 20/10/5/5; top-hat filters were applied with a filter size of 11/25/13/17, and Contrast Limited Adaptive Histogram Equalization (CLAHE) was conducted with a grid size of 11/7/7/7, using getStructuringElement(cv2.MORPH_RECT) or createCLAHE, respectively, from open-cv (Bradski, 2000).

On a related point, what is the interplay between contrast increase/denoising and image resolution (as this is a disadvantage of just using a Gaussian filter, for example)? And does the order of the MS- SSIM make a difference to resolution? I mainly ask this because in the microtubule data that is validated with the STED imaging (Figure 2A, lower row), the microtubule diameters appear thinner in the L2L 3S-SSIM image than the L1 image.

Thank you for this comment! We indeed observed an increase in resolution in the predictions after N2N and L2L training after using a MS-SSIM loss function. To quantify this effect, we extracted the FWHMs of 20 line profiles in the raw, predicted and processed images (see new **Figure S4**). We changed the text as follows:

old<br><br>262-265	A loss function-dependent trend is observable for both methods: using a $L_{MS\text{-}SSIM}$ instead of a L_1, the CNN learns (with decreasing M) to restore microtubules with higher contrast, especially when trained with images of two non-identical labels (see **SI Figure 2**).
new<br><br>252-257	A loss function-dependent trend is observable for both methods: using a $L_{MS\text{-}SSIM}$ instead of a L_1, the CNN learns (with decreasing M) to restore microtubules with increased sharpness, especially when trained with images of two non-identical labels (see **Fig. S2E**). This effect is quantifiable; extracted full width at half maxima (FWHMs) of line profiles across single microtubules in the images show that results obtained in L2L results are closest to microtubule diameters detected with STED microscopy (see **Fig. S4C**).

new<br><br>423-426	Further, the sharpening of structure with L2L after using a MS-SSIM loss function for the training – while advantageous for images of tubulin (see **Fig. S4C**) for which we know that the true microtubule diameter is not resolved with confocal microscopy - may be less desirable in images of other cell structures.

New<br><br>655-657<br><br>*methods*	The FWHM was derived from 20 randomly selected line profiles across single microtubules in images of tubulin, by averaging the line profile across 20 px and determining the Gaussian fit with scipy (Virtanen *et al.*, 2020) (see **Fig. S4C**).

<u>**Concern 3: Image artefacts:**</u> I have a concern with the data in the lower row of Figure 2A that all three deep learning methods appear to be collapsing structures onto one another (artificial sharpening) - this is most visible on the triangle-shaped crossing halfway up the image on the right-hand side, and on the two parallel microtubules running diagonally across the top left. It is really useful to have the STED validation here, as otherwise this sort of artefact would not be

© 2022. Published by The Company of Biologists under the terms of the Creative Commons Attribution License (https://creativecommons.org/licenses/by/4.0/).

obvious. In terms of biological information, although the input image clearly looks awful, I wonder whether this is still actually as reliable than the denoised data (e.g. if I had to manually trace the microtubules in both the input and L2L 3S-SSIM images, I think I would get results that I was equally confident in from both). When the authors mention hallucinations in images, it would be good to annotate examples of these either directly on the images or in a supplementary figure (to avoid obscuring the main figures too much). Examples are mentioned in lines 209-212 and lines 283-285, and it would be useful to guide the reader a little more here, especially if they are unfamiliar with looking at deep learning-generated images.

Thank you for this comment. We have made a few changes to the Figures and manuscript that aim to address this issue.

We have included a new **Figure S4A+B** that shows an example of those collapsing structures the Reviewer has mentioned. We changed the main text as follows:

old<br><br>257-259	The closer microtubules are packed in the cell, the less likely is the successful recovery of separate structure by the CNN as evident by comparing the results of both methods with the corresponding STED image (see **Figure 2A** (*bottom*)).
new<br><br>247-249	The closer microtubules are packed in the cell, the less likely is the successful recovery of separate structure by the CNN as evident by comparing the results of both methods with the corresponding STED image (see also **Fig. S4A,B**).

We annotated ROIs that show hallucination effects in **Figure S1A-C** and made the following change to the text:

old<br><br>206-208	On the other hand, the restored images after training the CNN with a *LMS-SSIM* exhibit cell structures with increasing sharpness, and erroneous predictions by the network occur (with lower *M*).
new<br><br>196-198	On the other hand, images after training the CNN with a *LMS-SSIM* exhibit cell structures with increased sharpness, and erroneous predictions by the network occur (with lower *M*) (see annotated ROIs in **Fig. S2A-C**).

Regarding post-processing/tracing microtubules in the images please see also our answer to comment 6.

Concern 4: Implementation/deployment of algorithm: Is the code freely available for people to download and use on their own data? Would any of the trained networks here be directly applicable to someone else's data, or should new networks be trained for each use instance? If the code is not available, or would not be practical to be used directly on fresh data from other sources, I personally think it is acceptable to just say that the work here is a useful proof of principle (as the manuscript is currently presented I am not sure if this is something that I can instantly use myself or not). If this method is already intended to be used by researchers, then there should also be guidance on which loss function is best to use – should this be a purely metric-based decision as shown in Table 1, or more subjective and based on the user's judgement (e.g. as in lines 335-337, also discussed in lines 435-436)?

Thank you for raising this point. The CARE network that we used for the N2N/L2L training in this work is actively maintained on github and includes multiple examples how to use the code. We included the link in the methods. Using it for the readers own data would simply require the implementation of a MS-SSIM loss function into this framework which is available in Tensorflow. Since fluorescence images can vary a lot regarding image noise, resolution, sample quality, etc.,

© 2022. Published by The Company of Biologists under the terms of the Creative Commons Attribution License (https://creativecommons.org/licenses/by/4.0/).

we do not recommend using our trained network for different images.

We changed the text as follows to address the concern:

old<br><br>166-169	We show that by introducing systematic sample differences in the training data a CNN can be successfully trained to reject not only image noise but also diffuse, label- dependent cytosolic signals in IF images that, in practice, decrease the contrast of a target structure.
new<br><br>158-161	We show proof-of-principle that by introducing systematic sample differences in the training data a CNN can be successfully trained to reject not only image noise but also diffuse, label-dependent cytosolic signals in IF images. Both can decrease the contrast of a target structure significantly in practice.

Comment 1: The description of CycleGANs (lines 60-62) is a little confusing, especially for a biological audience (e.g. what is back-translation?)

We thank the Reviewer for highlighting this, and we have reworded this section to make this argument more clearly. Please see below:

Old<br><br>60-62	CycleGANs allow the training with unaligned image pairs, addressing the "hallucination problem", - the introduction of artificial features in the generated images, - when training a classical GAN with unpaired data by implementing an additional training instance in which a second GAN is employed for back-translation [11,13].
new 57-62	The CycleGAN architecture addresses the "hallucination problem" which is the introduction of artificial features in generated images that is often observed when training a classical GAN (Zhu *et al.*, 2017). Here, a GAN is first trained to generate a higher quality image based on a corrupted input. Then, the generated image is fed into a second GAN that translates it back into the original image (back-translation), giving the network less freedom to make changes to an input (Zhu *et al.*, 2017; Lim *et al.*, 2020).

Comment 2: In the description of noise2clean (lines 95-99) it might be worth also highlighting reference 8 by the name 'CARE', as I think this is the name by which most people know it as.

Thank you for highlighting this issue. We made the following changes to reference other work and explain this more clearly:

old<br><br>95-97	L2L is also different to restoration methods with a *noise2clean* approach where a clean benchmark is required to train a network [8].
new 96-97	L2L is also different to restoration methods with a *noise2clean* approach where a clean benchmark is required to train a network (Goodfellow *et al.*, 2014; Weigert *et al.*, 2018; Wang *et al.*, 2019).

old<br><br>106-107	We selected the CSBDeep framework for the training that was previously used for CARE of noisy or under-sampled fluorescence images [8].

© 2022. Published by The Company of Biologists under the terms of the Creative Commons Attribution License (https://creativecommons.org/licenses/by/4.0/).

new<br><br>110-111	We selected the CSBDeep framework for the training that is also known as CARE network (Weigert *et al.*, 2018).

Also, I did not quite follow the argument against using noise2clean in IF data with non-dynamic specimens and high photon counts.

We thank the Reviewer for highlighting this. We have reworded this section to hopefully make the argument clearer. Please see below:

old<br><br>98-99	For *noise2clean*, the training data is generated by acquiring two images of the same label with, for example, varying exposure time, frame averaging or sampling density. Notably, this approach is rarely feasible in IF microscopy where cell specimens are fixed, and commercially available markers are efficient and comparably photo-stable, allowing the acquisition of images with high photon counts. Further, generating the necessary image pairs for the network training is time-consuming and significantly complicated by stage drift, overall resulting in a low benefit-cost ratio.
new<br><br>97-105	For *noise2clean*, the training data is generated by acquiring two images of the same label with, for example, different exposure time, sampling density or frame averaging. Notably, training a network to restore IF images that are acquired with low exposure time or sampling density is rarely feasible, since cell specimens are fixed, and commercial antibodies are comparably efficient and photo-stable, allowing the image acquisition with both parameters optimised right away. Generating image pairs to train a network to restore images acquired with low frame averaging, however, is time- consuming, and further complicated by stage drift and photo-bleaching, overall resulting in a low benefit-cost ratio.

Comment 3: In Table 1, evaluation metrics such as NRMSE can sometimes be a bit misleading because of good matching between large regions of background which dominate an image, rather than the structure itself.

We thank the Reviewer for this comment. We noticed this issue as well. For that reason, we only calculated those metrics for image patches that were generated with the "create_patches" function of the CARE/CSBDeep Framework. This function disregards background areas in the raw images. We amended the caption of **Table 1** to clarify this to the reader.

old<br><br>caption for Table 1	Average metrics were calculated between the respective training inputs or restored images of the input, respectively, (…) and the corresponding training benchmarks, using the image patch pairs that were excluded from the training for the validation (see **Table 2**).
new<br><br>caption for Table 1	Average metrics were calculated between the respective training inputs or restored images of the input, respectively, (…) and the corresponding training benchmarks. For that, the image patches that were used for the validation during the training were utilised that were created from non-background areas in the raw images (see **Table 2**).

SSIM lends itself to creating a map of similarity via its sliding window implementation – would it be possible for one of the datasets to show such a map (in addition to the RMS map as in Figure 1) given the emphasis on using MS-SSIM?

© 2022. Published by The Company of Biologists under the terms of the Creative Commons Attribution License (https://creativecommons.org/licenses/by/4.0/).

We thank the Reviewer for this helpful suggestion. We now include RMS and SSIM maps between the input and predicted images after L2L training for all datasets in new **Figure S1**. We made the following changes to the text:

old<br><br>198-200	With L2L, high intensity punctate regions are selectively filtered out as evident in the RMS maps between the raw images of AC-15 and the L2L results (see **Figure 1D** (*right*)).
New<br><br>188-190	The RMS maps between the raw images of AC-15 and the L2L results reveal a selective removal of high intensity punctate regions (see **Fig. 1D** (*right*); for further maps see **Fig. S1** in the Supplementary Information).

Comment 4: Is there a difference if you switch the input and benchmark images in training, or is the method somewhat commutative? If there is a difference, and the two labels are not of markedly different quality (as appears the case at least for the microtubules data), could this switching be used as an additional data augmentation? In my experience, many different markers/antibodies for the same structure have been similarly mediocre (i.e. it wouldn't be clear which to use as the benchmark).

We thank the Reviewer for this suggestion. We tried this by training the networks for different scenarios (label 1/2 as input/benchmark, label 2/1 as input/benchmark, label 1+2/2+1 as input/benchmark). In no scenario did we obtain better results than we have shown in our manuscript. While, qualitatively, some label pairs do look similar (e.g. tubulin), we did calculate different RMS and Michelson contrast values for all label pairs, and we do think these contrast values are a good indicator how best to train a CNN for L2L.

To address this issue, we added the following into the discussion:

New<br><br>466-469	We found that the calculated RMS and Michelson contrast values for images of two labels were good indicators to assign labels to "input" and "benchmark". Here, training a CNN with the reverse order or pairing the labels in both directions resulted in either worse or comparable prediction success.

Comment 5: I did not understand lines 424-426, sorry (what is a non-dynamic image corruption?)

We have now changed the phrasing of this part of our manuscript, and we hope that this clarifies the issue.

old<br><br>424-426	Also, artefacts were introduced that likely originated from non-dynamic image corruptions by the imaging system that were present in both noise realisations of a sample (see **Figure 2C** and **SI Figure 3B**).
new 411-<br><br>414	Also, some N2N results exhibited artefacts that might originate from static image corruptions introduced by the imaging system itself, which then would be present in both noise realisations of a sample (see **Fig. 2C** and **Fig. S5B**).

Comment 6: To show that L2L is useful, it would be good to actually demonstrate the binarization discussed in lines 466-467 in a Supplementary Figure, comparing L2L with just e.g. a big Gaussian blur or threshold. This would be the cherry on the cake to demonstrate how L2L can be used to help downstream analysis rather than just make more visually appealing images.

Thank you for making this suggestion! We now include the **new Figure S8** in which we show for an example image pair of each dataset how L2L compares to a Gaussian blur and N2N to generate a distance map or binary image. We modified/added to the text as follows:

© 2022. Published by The Company of Biologists under the terms of the Creative Commons Attribution License (https://creativecommons.org/licenses/by/4.0/).

old<br><br>465-467	Instead, L2L could serve as image pre-processing step to extract the binary information about the location of a specific structure in a cell image.
new<br><br>456-458	Instead, L2L could serve as image pre-processing step to extract the binary information about the location of a structure in the cell (see examples in **Fig. S8**).

new<br><br>658-666<br><br>*methods*	To generate distance maps or binarised images (see **Figure S8**), the following pre-processing steps were undertaken using above mentioned functions: for images of actin, a rolling-ball BG subtraction (radius=10), a top-hat filter (filter size=7) and CLAHE (tile size 11) were applied; for images of tubulin, a rolling-ball BG subtraction (radius=10) and a top-hat filter (filter size=11) were applied; for images of caveolae, a Gaussian filter (sigma=0.75) and a rolling-ball BG subtraction (radius=5) were applied; for images of PXN, a rolling-ball BG subtraction (radius=5) was applied. Lastly, objects below a size of 20 px (caveolae)/50 px (all else) were removed. Binary images were generated using the 75th/60th/93th/90th percentile as threshold for images of actin/tubulin/caveolae/PXN. Distance maps were generated using scipy (Virtanen *et al.*, 2020).

Our response to comments from reviewer 2:

Advance Summary and Potential Significance to Field:

The key advance or contribution of this paper is that the authors demonstrate a new application of standard CNN models which can help assist or accelerate quantitative cell biology findings.

Comments for the Author:

In general, I think the paper is well written and indeed demonstrates a new application of a CNN in helping accelerate quantitative cell biology study.

We thank the Reviewer for the kind feedback.

But, I do have two major concerns, which in my opinion must be addressed before publication.

Major concern 1: The paper's claim on what the model is actually doing is not accurate, and need some re-wording. The paper claims that "..., but current image restoration methods cannot correct for background signals originating from the label. Here, we report a new method to train a CNN as content filter for non-specific signals in fluorescence images that does not require a clean benchmark, using dual-labelling to generate the training data". But, there is no strong evident showing the proposed L2L model actually correct the background signals originating from labels and only filter out the target content. What the L2L model actually does is simply a "style transfer", namely transferring the image from one type of signals or styles to another, via the training using dual-labelling images. For example, the paper mentions that "While the phalloidin stain labels almost exclusively the actin filaments, images of the antibody (AC-15) exhibit a high background signal in the cell body. This background signal likely originates from unspecific binding and/or binding to cytosolic protein by the AB, resulting in high intensity punctate regions as observed in the cell cytoplasm". We can see that the model is simply transferring one type of signal (AC-15) with high background signal to another type of signals (phalloidin) with much less background signals. The background signals or noise still exist, but just transformed from one type of background signal to another. I would suggest just to avoid explicitly claiming filtering out the structure signal or correcting background signals, instead saying something like transferring one label to another to make the structure more visible or something similar.

© 2022. Published by The Company of Biologists under the terms of the Creative Commons Attribution License (https://creativecommons.org/licenses/by/4.0/).

Thank you very much for making this valid point. We agree that the principle of L2L is the style transfer from one label to the other. However, we would make the argument that our results do not only show a style transfer after L2L training. For instance, image noise is not transferred but removed by a CNN after L2L training, and intensity fluctuations visible in the reference images for the tubulin dataset (see **Figure S2D-F**) are not translated in the predictions – following the principle of N2N where non- predictable signals are removed by a CNN. Further, images used as benchmark for L2L training are predicted with higher structural contrast by a trained CNN also (see **Figure 3D** and Figure **S8B,D,F,H**), although they already match the target style. This is different to the results obtained with the CycleGAN where, as the Reviewer points out, noise is transferred from "one style to the other" and not removed.

We hope that the following changes made to the text address the concern:

old<br><br>81-94	We propose a new application of DL in fluorescence microscopy where a neural network is trained as content filter of label-induced unspecific cytosolic signals in fluorescence images of cellular structures. We call this method *label2label* (L2L). For L2L, a CNN is trained with image pairs of cells that were dual-labelled for the same distinct cellular structure of interest. L2L utilises the varying performance of antibodies and stains in IF microscopy. We hypothesized that a CNN trained with two images of a cellular structure that originate from two non-identical labels and therefore exhibit sample differences would act as a content filter - where fluorescence signals that systematically vary in the images are rejected, while correlating, structural signal is restored. Therefore, L2L is different to N2N. In both methods a network is trained without clean benchmark images, but in L2L differences between the training input and benchmark images are not only originating from dynamic image corruptions like noise, but also inherent sample (=label) differences. Consequently, fluorescence signals from cytosolic protein and unspecific binding that, in practice, lower structural contrast in IF images are retrieved in restorations after N2N training, whereas a network acts as filter of such image content after L2L training when selecting appropriate training data.
new 80-95	We propose a new application of DL in fluorescence microscopy where a neural network is trained to significantly reduce label-induced unspecific cytosolic signals in fluorescence images of cellular structures. We call this method *label2label* (L2L). For L2L, a CNN is trained with image pairs of cells that are dual-labelled for the same distinct cellular structure of interest. L2L utilises the varying performance of antibodies and stains in IF microscopy. We hypothesized that a CNN trained with two images of a cellular structure that originate from two non-identical labels would act content filter- like - where fluorescence signals that systematically vary in the images are rejected, while correlating, structural signal is restored. Here, the underlying principle of L2L is a so-called style transfer where a neural network is trained to merge the content in input images with the style of reference images (Jing *et al.*, 2020). Since input and benchmark images highly correlate, L2L is also comparable to N2N. In both methods a network is trained without clean benchmark images, however, in L2L differences between the training images are not only originating from dynamic image corruptions like noise, but also inherent sample (=label) differences. Consequently, fluorescence signals from cytosolic protein and unspecific binding that, in practice, lower structural contrast in IF images are retrieved in restorations after N2N training, whereas a network reduces such signals after L2L training when selecting appropriate training data.

old<br><br>198-200	With L2L, high intensity punctate regions are selectively filtered out as evident in the RMS maps between the raw images of AC-15 and the L2L results (see **Figure 1D** (*right*)).

© 2022. Published by The Company of Biologists under the terms of the Creative Commons Attribution License (https://creativecommons.org/licenses/by/4.0/).

new<br><br>188-190	The RMS maps between the raw images of AC-15 and the L2L results reveal a selective removal of high intensity punctate regions (see **Fig. 1D** (*right*); for further maps see **Fig. S1** in the Supplementary Information).
old<br><br>204-206	For both methods, using a *L1* for the training leads in comparison to more conservative predictions, where, with L2L, non-filamentous signal is filtered out by the network, but actin filaments appear relatively blurry.

new<br><br>193-196	For both methods, using a *L1* for the training leads in comparison to more conservative predictions, where, with L2L, non-filamentous signal is reduced by the network, but actin filaments appear relatively blurry.

old<br><br>276-277	We find that the CNN performance as content filter for caveolae after N2N and L2L training is highly dependent on the training loss function (see **Figure 2C**).
new<br><br>268-269	We find that the CNN performance after N2N and L2L training is highly dependent on the training loss function (see **Fig. 2C**).

old<br><br>280-283	Here, cytosolic background signal in the image is successfully filtered out by the network after L2L training, resulting in restorations with higher sample-to-background ratios than the corresponding 20-frame average STED images of both labels (see **Figure 2** and **SI Figure 3**).
new 272-<br><br>275	Here, cytosolic background signal in the image is clearly reduced by the network after L2L training, resulting in restorations with higher sample-to-background ratios than in corresponding 20-frame average STED images of both labels (see **Fig. 2** and **Fig. S5A- C**).

old<br><br>321-323	To test if an artificial network can also be trained as content filter with unpaired L2L data, a CycleGAN was trained and its performance was compared to the CNN.
new<br><br>312-314	To test if an artificial network can also be trained with unpaired L2L data, a CycleGAN was trained and its performance was compared to the CNN.

old<br><br>340-342	In addition, we find that the trained CNN acts as content filter of cytosolic background signal in the training benchmark (Y113) with an enhanced signal-to-background ratio, although these images were not used as input for L2L training (see **Figure 3D**).
new<br><br>332-334	In addition, we find that the trained CNN reduces cytosolic background signal in the training benchmark (Y113) as well, although these images were not used as input for L2L training (see **Fig. 3D**).

© 2022. Published by The Company of Biologists under the terms of the Creative Commons Attribution License (https://creativecommons.org/licenses/by/4.0/).

old<br><br>344-346	Lastly, the ability of a CNN to filter out cell features in IF images based on their spatial intensity distribution was tested by training a network to separate superposed confocal images of two different cellular targets.
new<br><br>336-337	Lastly, the ability of a CNN to transfer style between IF images with correlating structural signal was tested by training a network to separate superposed confocal images of two different cellular targets.

old<br><br>390-391	We show that a CNN can be successfully trained to filter out unspecific, label-induced fluorescence signals (…).
new<br><br>382-383	We show that a CNN can be successfully trained to reduce unspecific, label-induced fluorescence signals (…).

old<br><br>402-403	The network performance as content filter was dependent on the level of correlation between the images of the two labels and the training loss functions.
new<br><br>391-392	The network performance is dependent on the level of correlation between the images of the two labels and the training loss functions.

old<br><br>409-411	Here, the to-filter-out signal deviated sufficiently between the images, allowing the network to clearly distinguish to the cellular structure that correlated for both labels during L2L training.
new<br><br>398-399	Here, unspecific background signals differed sufficiently between the images of both labels.

old<br><br>446-448	As expected, the evaluation of repeated 8/10-fold cross-validations (…) showed that using a high number of image pairs to train the CNN as content filter is advisable (see **Figure 5**).
new<br><br>438-440	As expected, the evaluation of repeated 8/10-fold cross-validations (…) showed that using a high number of image pairs to train a CNN for the style transfer between two labels is advisable (see **Fig. 5**).

© 2022. Published by The Company of Biologists under the terms of the Creative Commons Attribution License (https://creativecommons.org/licenses/by/4.0/).

old<br><br>496-506	We present a new DL-based image restoration method for images of cellular structures that utilises the varying performance of labels in immunofluorescence microscopy. We show that by training a CNN that was developed for CARE, with images of two non- identical labels that target the same cellular structure but exhibit systematic sample differences, the network learns to selectively restore the correlating signal in the images. Like other methods, L2L relies on the convention of the network to under- estimate inherently unpredictable signal. However, with L2L, not only image noise but also label-induced fluorescence signal in the cell specimen can be removed in the images after selecting appropriate training data. The ability to correct images for unspecific binding, inhomogeneous labelling of a structure or binding to cytosolic protein makes L2L, to our knowledge, unique in comparison to other deep learning-based image restoration methods that are currently used in cell biology.
new<br><br>493-502	We present a new DL-based image restoration method for images of cellular structures that utilises the varying performance of labels in IF microscopy; we call this method L2L. With L2L, we show that by training a CNN for a style transfer between two non-identical labels of a shared target, the network can be systematically trained to reduce unspecific cytosolic background signals and enhance structural contrast in IF images. Like other methods, L2L relies on the convention of the network to under-estimate inherently unpredictable signal. However, with L2L, not only image noise but also label- induced fluorescence signals in the cell specimen can be reduced in the images after selecting appropriate training data. The ability to significantly lower unspecific binding, inhomogeneous labelling of a structure or binding to cytosolic protein in IF images makes L2L, to our knowledge, unique in comparison to other DL-based image restoration methods that are currently used in cell biology.

Major concern 2: Are all structures able to be imaged via dual-labelling images? If not, I think this needs to clearly noted in the paper and more importantly claimed as one disadvantage over other methods like N2N which has no such requirement.

We thank the reviewer for this comment. The image pairs of all structures for the network trainings in our work were generated via dual-labelling of the cellular structure. The sample preparation is relatively straightforward thanks to secondary antibody labelling and a number of commercially available antibody combinations. However, not all label pairs are suitable to generate training data for L2L (there has to be an observable difference in both images), while for N2N (noisy) images of any label can be used to train a CNN. Therefore, we add the following to the manuscript:

new<br><br>463-466	However, contrary to N2N, L2L requires the sample preparation with two markers that exhibit systematic differences in the respective images to allow training for a useful style transfer between labels. Therefore, not all label pairs of a target structure are suitable to generate the necessary training data.

Minor concern 1: why CycleGAN is only tested on FA structures?

In the paper, we only show the results for the PXN dataset since they are the best datasets highlighting the advantages obtained using the L2L datasets when training a CycleGAN with unpaired image patches. We recognise that the other results are of interest for the reader as well, these are now **added as Figure S6.** We also made the following changes to the text:

© 2022. Published by The Company of Biologists under the terms of the Creative Commons Attribution License (https://creativecommons.org/licenses/by/4.0/).

old<br><br>107-110	Moreover, for one dataset, we trained a CycleGAN with unpaired images of the two labels to assess if, in principle, a network can also be trained as content filter using IF images that stem from two different datasets [11].
New<br><br>111-113	Moreover, we trained a CycleGAN with unpaired images to assess if, in principle, a network can also be trained with IF images that stem from two different datasets (*Zhu et al.*, 2017).

new<br><br>314-315	Further results for other datasets are shown in **Fig. S7**.

old<br>471-477	Further, the use of unpaired training data was explored by training a CycleGAN with the unaligned images of the label pairs generated in this work (see **Figure 3A+C** and **Figure S6**). While the restored images of a CycleGAN after L2L training showed a decrease in background signal, which in the PXN dataset originated from cytosolic protein, the results were not comparable to the restorations obtained after training a CNN. We found that, since the generator network in the CycleGAN is trained to fool a discriminator based on a noisy benchmark (Y113), artefacts were introduced by the CycleGAN. This might be avoidable when training with cleaner reference images in the future.
new<br><br>470-476	We also trained a CycleGAN with unaligned label pairs of a target structure (see **Fig. 3A**,**C** and **Fig. S7**). While the generated images of a trained CycleGAN exhibited reduced unspecific cytosolic signals, it was outperformed by a trained CNN. Since the generator in the CycleGAN is trained to fool a discriminator based on noisy benchmarks, either little to no change to the input image was observed (tubulin/caveolae) or artefacts were introduced (actin/PXN) by the network to match the style of the reference image. Prior denoising of the images via Gaussian filtering led to slightly better results (see **Fig. S7**). A higher performance might be achieved with cleaner reference images.

old<br><br>641-645	The CycleGAN was trained with unaligned images of the PXN dataset (…). Training was conducted with a batch size of 4, an epoch number of 4 (3 with linear decay of the learning rate) and a scaling factor of 0.0005 for the network initialization.
new<br><br>639-644<br><br>*methods*	The CycleGAN was trained with unaligned images of the actin, tubulin, caveolae and PXN dataset (…). Training was conducted with a batch size of 4, an epoch number of 4/10 (3/9 with linear decay of the learning rate) for the PXN/other dataset(s) and a scaling factor of 0.0005 for the network initialization.

Minor concern 2: I would highly recommend to show some not quite successful ROI in the predictions. For example, in Figure 1, I can clearly see some not quite good ROI in the predictions, but the zoom- in areas are more like good ROI examples.

Thank you for this comment. Part of this concern was also shared with Reviewer 1. We have copied our answer below.

We have included a **new Figure S4A+B** that shows an example of collapsing microtubule structures in the predictions in comparison that the high resolution STED image. We changed the main text as follows:

© 2022. Published by The Company of Biologists under the terms of the Creative Commons Attribution License (https://creativecommons.org/licenses/by/4.0/).

old<br><br>257-259	The closer microtubules are packed in the cell, the less likely is the successful recovery of separate structure by the CNN as evident by comparing the results of both methods with the corresponding STED image (see **Figure 2A** (*bottom*)).
new 247-<br><br>249	The closer microtubules are packed in the cell, the less likely is the successful recovery of separate structure by the CNN as evident by comparing the results of both methods with the corresponding STED image (see **Fig. 2A** (*bottom*) and **Fig. S4A,B**).

Further, we annotated ROIs that show hallucination effects in **Figure S2A-C** and made the following change to the text:

old<br><br>206-208	On the other hand, the restored images after training the CNN with a *LMS-SSIM* exhibit cell structures with increasing sharpness, and erroneous predictions by the network occur (with lower *M*).
new 196-<br><br>198	On the other hand, predictions by a CNN after training with a LMS-SSIM exhibit cell structures with increased sharpness, and erroneous predictions by the network occur (with lower M) (see annotated ROIs in **Fig. S2A-C**).

Minor concern 3: I would highly recommend to do another type of validation. Specially, I would recommend to do segmentation on the cleaner images and the predictions, as well as the noisier images to show that after restoration the images can be better segmented to permit more accurate downstream quantitative analysis. For example, for the actin example, segmentation from AC-15 can be hard, but segmentation from Phalloidion might be much easier so that the actual topology of the actin structures can be better identified. It would be good to show the segmentation from prediction is comparable to segmentation from Phalloidion and better permits accurate downstream analysis comparing to AC-15.

Thank you for making this suggestion. Part of this concern was also shared with Reviewer 1. We have copied our answer below.

We now include the **new Figure S8** in which we show for an example image pair of each dataset how L2L compares to a Gaussian blur and N2N to generate a distance map or binary image. We modified/added to the text as follows:

old<br><br>465-467	Instead, L2L could serve as image pre-processing step to extract the binary information about the location of a specific structure in a cell image.
New<br><br>456-458	Instead, L2L could serve as image pre-processing step to extract the binary information about the location of a structure in the cell (see examples in **Fig. S8**).

© 2022. Published by The Company of Biologists under the terms of the Creative Commons Attribution License (https://creativecommons.org/licenses/by/4.0/).

new<br><br>658-666<br><br>*methods*	To generate distance maps or binarised images (see **Figure S8**), the following pre-processing steps were undertaken using above mentioned functions: for images of actin, a rolling-ball BG subtraction (radius=10), a top-hat filter (filter size=7) and CLAHE (tile size 11) were applied; for images of tubulin, a rolling-ball BG subtraction (radius=10) and a top-hat filter (filter size=11) were applied; for images of caveolae, a Gaussian filter (sigma=0.75) and a rolling-ball BG subtraction (radius=5) were applied; for images of PXN, a rolling-ball BG subtraction (radius=5) was applied. Lastly, objects below a size of 20 px (caveolae)/50 px (all else) were removed. Binary images were generated using the $75^{th}/60^{th}$/93th/90th percentile as threshold for images of actin/tubulin/caveolae/PXN. Distance maps were generated using scipy (Virtanen *et al.*, 2020).

<u>Other changes made to the article:</u>

1. Merging of (*old*) **SI Figures 1+2** and **3+5** to (*new*) **Figures S2** and **S5**
2. Added running title (32 characters):

new<br><br>title page	Title: Label2label: Training a neural network to selectively restore cellular structures in fluorescence microscopy<br><br>Running Title: Label2label

3. Cut key words to meet limit (6):

old<br><br>title page	Key words: image content filter, fluorescence microscopy, antibody labelling, deep learning, convolutional neural networks, content-aware image restoration, noise2noise, cellular structures, focal adhesions, actin cytoskeleton, microtubule network, caveolae
new<br><br>title page	Key words: convolutional neural networks, content-aware image restoration, antibody labelling, noise2noise, cellular structures, fluorescence microscopy

4. Added summary statement:

new<br><br>title page	Label2label is a new deep learning-based image restoration method that reduces cytosolic background signals in immunofluorescence images of cellular structures.

© 2022. Published by The Company of Biologists under the terms of the Creative Commons Attribution License (https://creativecommons.org/licenses/by/4.0/).

5. Cuts to abstract to meet word limit (180):

Abstract after changes<br><br>title page	Immunofluorescence (IF) microscopy is routinely used to visualise the spatial distribution of proteins that dictates their cellular function. However, unspecific antibody binding often results in high cytosolic background signals, decreasing the image contrast of a target structure. Recently, convolutional neural networks (CNNs) were successfully employed for image restoration in IF microscopy, but current methods cannot correct for those background signals. We report a new method that trains a CNN to reduce unspecific signals in IF images; we name this method *label2label* (L2L). In L2L, a CNN is trained with image pairs of two non-identical labels that target the same cellular structure. We show that after L2L training a network predicts images with significantly increased contrast of a target structure, which is further improved after implementing a multi-scale structural similarity loss function. Here, our results suggest that sample differences in the training data decrease hallucination effects that are observed with other methods. We further assess the performance of a cycle generative adversarial network, and show that a CNN can be trained to separate structures in superposed IF images of two targets.

6. Cuts to manuscript to meet word limit (8000):

old<br><br>62-64	Other examples include *noise2void*, an unsupervised method that removes camera shot noise (Krull, Buchholz and Jug, 2019), *DivNoising*, where a variational autoencoder is trained to restore a distribution of denoised images based on a noise model (Prakash, Krull and Jug, 2020), and *noise2noise* (N2N) (Lehtinen *et al.*, 2018).

new 62-64	Other examples include *noise2void*, an unsupervised method that removes camera shot noise (Krull, Buchholz and Jug, 2019), and *noise2noise* (N2N) (Lehtinen *et al.*, 2018).

© 2022. Published by The Company of Biologists under the terms of the Creative Commons Attribution License (https://creativecommons.org/licenses/by/4.0/).

Old<br><br>111-130	To establish and evaluate our method we generated image data across four different distinct cellular structures: the actin cytoskeleton, the microtubule network, and discrete plasma membrane structures, namely caveolae and focal adhesions. Actin is a conserved protein in eukaryotic cells that plays a major role in cellular functions like cell migration, cell motility or sustaining the cell shape (Dominguez and Holmes, 2011; Vedula *et al.*, 2017). It exists in two states: as globular (G-) actin in its monomeric form, or polymerised as filamentous (F-) actin (Suarez and Kovar, 2016). Evidence suggests that different actin isomers vary in function and localisation; they appear, for example, in stress fibres, circular bundles, cell-cell contacts or the cell cortex (Dugina *et al.*, 2009). Microtubules are fundamental cytoskeletal polymeric structures in all eukaryotic cells that, amongst others, impact cell transport, cellular signalling via cilia and cell division (Nogales, 2000). The inhibition and promotion of microtubule assembly has been shown to promote mitotic arrest which makes microtubules a prominent target in cancer therapy research (Pellegrini and Budman, 2005). Caveolae are plasma membrane invaginations composed of heterooligomeric CAVEOLIN and CAVIN protein complexes, and are abundant in many mammalian cell types (Hansen and Nichols, 2010; Khater *et al.*, 2018). Caveolae are multifunctional organelles that are implicated in transcytosis, lipid homeostasis and cellular signalling (Rausch and Hansen, 2020). Focal adhesions (FAs) are cellular membrane-associated multi-protein component biomechanical structures. FAs are integral in the ability for most cells to sense and respond to the extracellular matrix and physical changes in the cellular microenvironment (Martino *et al.*, 2018; Rausch and Hansen, 2020). The ability of a CNN to selectively restore distinct cellular structures in IF images after training with carefully selected image data was further assessed by training a CNN as separator of two markers in superposed IF images. For that, images were acquired of fixed cells that were dual-labelled with a nuclear marker and an antibody against the plasma membrane protein CD44 (see last results section).
new<br><br>114-121	To establish and evaluate our method we generated image data across four different distinct cellular structures: the actin cytoskeleton (Dugina et al., 2009; Suarez and Kovar, 2016), the microtubule network (Nogales, 2000; Pellegrini and Budman, 2005), and discrete plasma membrane structures, namely caveolae (Rausch and Hansen, 2020) and focal adhesions (Martino et al., 2018). The ability of a CNN to selectively restore distinct cellular structures in IF images after training with carefully selected data was further assessed by training a CNN as separator of two markers in superposed IF images. Here, images were acquired of fixed cells that were dual-labelled with a nuclear marker and an antibody against the plasma membrane protein CD44 (Ilangumaran, Borisch and Hoessli, 2010).

old<br><br>137-139	The most commonly used deviation-minimising estimators are the least absolute deviation loss function $L1$ (or LAD) and the least square deviation loss function $L2$ (…) where $\hat{y}$ (=$g\theta(xt)$) is the predicted image and N the total pixel number in the image.

new<br><br>128-130	Common loss functions are the least absolute deviation loss function $L1$ or the least square deviation loss function $L2$ (…) where $\hat{y}$ (=$g\theta(xt)$) is the predicted image and N the total pixel number.

© 2022. Published by The Company of Biologists under the terms of the Creative Commons Attribution License (https://creativecommons.org/licenses/by/4.0/).

old<br><br>150-156	To calculate the MS-SSIM index, a low-pass filter is applied to the image patches after each iteration (if M>1), followed by down-sampling by a factor of 2. This approach makes the MS-SSIM index sensitive to differing viewing conditions, such as differing perceived resolution from the point of observation.<br><br>The MS-SSIM index exhibits values between (-1, 1) where 0 implies no structural similarity and -1/1 a negative/positive correlation between two images. Since a CNN aims at minimising loss during training, for a MS-SSIM loss function ($L_{MS\text{-}SSIM}$) follows (…).
new 143-148	For the calculation, a low-pass filter is applied to the image patches after each iteration (if M>1), followed by down-sampling by a factor of 2, making the MS-SSIM index sensitive to differing viewing conditions.<br><br>The MS-SSIM index exhibits values between (-1, 1) where -1/0/1 imply a negative/no/a positive correlation between the images. To satisfy **Eqn. 1**, for a MS-SSIM loss function ($L_{MS\text{-}SSIM}$) follows (…).

old<br><br>160-163	For N2N training, the same pre-processing steps and network settings were applied, but two noise realisations of the same label were used as training input and benchmark instead. We wondered if using a $L_{MS\text{-}SSIM}$ instead of a $L1$ for N2N training would train a CNN to restore images not only with reduced image noise but also increased structural contrast.
new<br><br>153-155	For N2N training, the same settings were applied, but the network was trained with two noise realisations of the same label instead.

old<br><br>480-483	The use of CNNs as content filter in IF microscopy could increase possibilities for multiplex imaging in the future. For example, CNNs could be employed to separate two or more markers in cell images that were acquired with microscopy setups that have a limited number of excitations sources or detectors.
new 479-481	Our results show that CNNs could be utilised in the future to separate the fluorescence signals from multiple markers in microscopy images that were acquired with imaging setups that have a limited number of excitations sources or detectors.

old<br><br>487-489	For that, the image pairs for the network training can be generated post-image acquisition *in vitro*, by fixing the cells and labelling with a higher performing antibody against the target structure.
new 484-486	Training data can be generated post-image acquisition *in vitro*, by fixing the cells and labelling with a higher performing antibody against the target structure.

© 2022. Published by The Company of Biologists under the terms of the Creative Commons Attribution License (https://creativecommons.org/licenses/by/4.0/).

after changes<br><br>*acknowledge-ments*	L.S.K. was supported by the MRC and EPSRC for Doctoral Training in Optical Medical Imaging (EP/L016559/1). J.V. was supported by the Wellcome Trust (208345/Z/17/Z). STED imaging was performed at ESRIC, which is supported by the MRC and the Wellcome Trust. Work on-going in the Gram Hansen lab is supported by a University of Edinburgh Chancellor's Fellowship, by the Worldwide Cancer Research and the June Hancock Mesothelioma Research Fund. G.M. was supported by the grants MR/K015583/1, BB/P02565X/1 and BB/T011602/1. The authors thank Martin Weigert (EPFL, Switzerland) for his helpful feedback.

Second decision letter

MS ID#: JOCES/2021/258994

MS TITLE: Label2label: Training a neural network to selectively restore cellular structures in fluorescence microscopy

AUTHORS: Lisa Sophie Kölln, Omar Salem, Jessica Valli, Carsten Gram Hansen, and Gail McConnell
ARTICLE TYPE: Research Article

I am happy to tell you that your manuscript has been accepted for publication in Journal of Cell Science, pending standard ethics checks.

Reviewer 1

Advance summary and potential significance to field

My opinion on the advance made in this paper and its potential significance to the field remains the same as prior to receiving the revisions.
I think that this method is a useful demonstration of how deep learning image restoration can be applied to a very cell biology-specific problem. It will hopefully provoke more thought in the field of how we can tailor advanced image restoration techniques to real challenges faced by experimental biologists.

Comments for the author

The authors have sufficiently addressed all of my comments and concerns in their response and the revised manuscript. I was particularly impressed by the work that authors put into exploring weights and filters at my request (and I agree that this is not necessary to include in the revised manuscript - while I personally find these fascinating, I don't think many other readers would!).
Signed, Siân Culley

Reviewer 2

Advance summary and potential significance to field

The authors did a good job to address all my concerns.

Comments for the author

No more concern

© 2022. Published by The Company of Biologists under the terms of the Creative Commons Attribution License (https://creativecommons.org/licenses/by/4.0/).
